# Class Separation Improvements in Pixel Classification Using Colour Injection

**DOI:** 10.3390/s100807803

**Published:** 2010-08-20

**Authors:** Edward Blanco, Manuel Mazo, Luis Bergasa, Sira Palazuelos, Jose Rodríguez, Cristina Losada, Jose Martín

**Affiliations:** 1 Department of Electronics and Electromechanics, Pontificia Universidad Católica Madre y Maestra, 822 Santiago, Dominican Republic; E-Mail: eblanco@pucmmsti.edu.do (E.B.); 2 Electronics Department, University of Alcalá, Campus Universitario s/n, 28805, Alcalá de Henares, Spain; E-Mails: mazo@depeca.uah.es (M.M.); bergasa@depeca.uah.es (L.B.); jmra@depeca.uah.es (J.R.); losada@depeca.uah.es (C.L.); jlmartin@depeca.uah.es (J.M)

**Keywords:** pixel classification, colour clustering, colour segmentation, class separation, colour sub-spaces, colour injection

## Abstract

This paper presents an improvement in the colour image segmentation in the Hue Saturation (*HS*) sub-space. The authors propose to inject (add) a colour vector in the Red Green Blue (*RGB*) space to increase the class separation in the *HS* plane. The goal of the work is the development of an algorithm to obtain the optimal colour vector for injection that maximizes the separation between the classes in the *HS* plane. The chromatic Chrominace-1 Chrominance-2 sub-space (of the Luminance Chrominace-1 Chrominance-2 (*YC_1_C_2_*) space) is used to obtain the optimal vector to add. The proposal is applied on each frame of a colour image sequence in real-time. It has been tested in applications with reduced contrast between the colours of the background and the object, and particularly when the size of the object is very small in comparison with the size of the captured scene. Numerous tests have confirmed that this proposal improves the segmentation process, considerably reducing the effects of the variation of the light intensity of the scene. Several tests have been made in skin segmentation in applications for sign language recognition via computer vision, where an accurate segmentation of hands and face is required.

## 1. Introduction

In recent years, a significant amount of work has been published in the field of colour segmentation for Human Computer Interfaces (*HCI*). We would like to emphasize those related to the segmentation of the natural colour of skin. In this area, Phung *et al.* [1] proposed a skin segmentation method using a Bayesian classifier, obtaining satisfactory results for different colour spaces such as: *RGB*, Hue Saturation Value (*HSV*), Luminance blue-Chrominance red-Chrominance (*YC_b_C_r_*) and Commission Internationale de l'Éclairage’s Luminosity a-channel b-channel (*CIE-Lab*), even under adverse illumination conditions. Hsu *et al*. [2] suggested the detection of face skin considering a nonlinear subspace from the *YC_b_C_r_* space to partially compensate the luminosity variations.

The robustness of the segmentation against luminosity changes is one of the most desirable features in colour segmentation systems. For this reason, much work on this topic has been focused on minimizing the effects of illumination changes by using colour spaces where the luminance or intensity component can be easily isolated, thus providing chromatic constancy. The actual trend in applications with important time-varying-illumination changes is to use dynamic colour models that can adapt themselves to compensate for variations of the scene illumination. In this area, an extensive overview of previous investigations in the skin colour segmentation field is presented by Sigal *et al*. [3].

The most frequently used colour spaces in these types of applications are *HSV* [3,4] and normalized Red Green (*rg*) [5–7]. The *HSV* space, as well as the Hue Saturation Intensity (*HSI*) and Hue Lightness Saturation (*HLS*) spaces, are widely used in image processing because it is very intuitive for the human brain to interpret the information as it is represented. In some works, only the Hue (*H*) and Intensity (*I*) components are used in the clustering process [8]. In other cases, a threshold value for the Saturation (*S*) of each pixel based on its intensity is defined [9]. This threshold is used before the clustering process to determine if *S* should be replaced by *H* or *I*.

In general, all these segmentation proposals offer good results for objects with significant size in the scene or in cases where the main goal is object tracking, but not in the case of shape recognition. If the goal is to recognize the object shape, the system requirements are higher and very accurate segmentation techniques should be applied. Further difficulties may arise if the images have low quality and spatial resolution. Sign language recognition systems based on computer vision are a good example of these types of applications. In this case, the camera should capture all the upper parts of the speaker’s body, implying that the parts to segment (hands and face) constitute a small part of the captured scene. In this field, Habili *et al*. [10] performed a pixel-by-pixel classification of the skin colour with discriminant features of the *C_b_C_r_* plane, using the Mahalanobis distance, but they needed a fusion of motion cues to obtain good results. Similar skin segmentation is achieved in the work done by Chai *et al*. [11], where post-segmentation stages were applied, such as morphological operations, in order to surpass the limitations of the segmentation. The *YC_b_C_r_* space has been also used [11]. This colour space is one of the most widely used in the segmentation process.

In this field, Ribiero and Gonzana [12] presented hand segmentation in video sequences by means of the Gaussian Mixture Model (GMM) background subtraction algorithm, which is a well-known statistical model for density estimation due to its tractability and universal approximation capability. In this work, [12], an adaptive Gaussian mixture in time is used to model each pixel distribution in *RGB* space. In Huang and Liu’s work [13], clustering of colour images using GMM technique in *HSV* space is performed.

Less common colour spaces are also used in other works: both linear transformation spaces, like Luminance E-channel S-channel (*YES*) [14], and non-linear, spaces like the Uniform Chromaticity Scale (*UCS*) spaces, such as Luminance u-channel v-channel (L*u*v*) and its representation in cylindrical coordinates Intensity Hue Saturation (*IHS*) [15], Saturation Tint Value (*STV*) [16] which is a representation of *HSV* space by the normalized *RGB* components. Other spaces used are the Spherical Coordinate Transform (*SCT*) [17] and the geodesic chromaticity space *pq* [18].

We can also find works related to object/background segmentation with the objective of efficiently delimiting object edges. Some of these publications present the use of graph cuts in N-dimensional images to segment medical images from computed tomography (CT) scanners [19,20], and multilevel graph cuts to accelerate the segmentation and optimize memory use [21]. From our point of view, the main disadvantage of these works is that they are not designed for real-time purposes.

The conclusion of these previous works is that important unresolved problems still exist in order to obtain efficient skin segmentation, especially if we take into account that many applications require real time processing, include complex scenes, are prone to important illumination changes, and the objects to segment (face, arms and hands) are small when compared to the captured scene.

Our contribution to the solution of this segmentation problem is to use an object/background pre-processing technique to enhance the contrast (in the *HS* plane) between the colours corresponding to the objects to segment and the background in each frame. This pre-processing consists of increasing the separation between the object and background classes in the *HS* plane to optimize the segmentation in that plane.

In our proposal, to increase the class separation, a colour vector of components Δ*_R_*, Δ*_G_*, Δ*_B_*, is added to the *R*, *G* and *B* images directly captured from the camera, modifying the value of each pixel (*n*) to (*R_n_*+Δ*_R_*, *G_n_*+Δ*_G_*, *B_n_*+Δ*_B_*). The objective of this paper is to present the process needed to obtain the values Δ*_R_*, Δ*_G_* and Δ*_B_* that optimize the separation between the classes of interest once the image has been converted to the *HS* plane. This optimization is carried out by means of an algorithm that maximises the Fisher Ratio. We have called the colour vector addition process “colour injection”. In our proposal, the colour injection process is achieved using the relationships between the *RGB*, *YC_1_C_2_* [8,22,23] and *HSI* [24–26] colour spaces, and the properties of the *C_1_C_2_* plane.

Our system may be particularized to recognize sign language in real-time and special attention has been paid to the detection of the geometric form of the parts to segment, hands and face edges, in each frame. Our proposal has been thoroughly tested with very good results even with illumination variations, because it isolates the *I* component. We always attempt to work outside the instability or achromatic zone of the *HS* plane, due to the convenient redistribution in the *HS* plane of the existing classes in the colour injected image (seen in [15] for the *IHS* space). In order to perform a comparative qualitative study between the segmentations of the original images and the colour injected images (proposal presented in this paper), a GMM clustering technique in the *HS* classification domain is used. This technique has been used in a similar way for the *HSV* space [13]. In previous works, different formulations for the *HSI* space can be found [22,24–26]. We use the formulation proposed in [26].

This paper has been organized as follows: Section 2 describes the basis of the proposed algorithm to increment the separation between classes. Section 3 presents the criteria considered when separating the classes. Section 4 describes the off-line initialization stage of the proposed algorithm. Section 5 details how to improve the separation between classes in the *HS* plane starting from their location in the *C_1_C_2_* plane. Section 6 presents the algorithm that performs the optimal class separation. Section 7 describes how to obtain the colour vector for injection, and its effects in the captured images. Section 8 contains the experimental results, and Section 9 provides the conclusions and future work.

## 2. Overview of the Colour Injection Algorithm

The objective of this work is to improve the segmentation process using colour injection. In order to do that, a colour vector for injection is obtained for each captured image in the *RGB* space. This colour vector is considered optimal, because it is calculated to maximize the separation between the classes to segment in the *HS* plane (subspace where the segmentation is performed). For this reason, this colour vector will be called optimal colour vector in this paper and will be denoted by **i_r_**. It is injected in the *RGB* space and is calculated starting from significant samples (seeds) from the object to segment and from the part of the scene considered as background. The procedure to obtain the vector **i_r_**, and the reason why it is optimal is explained in Sections 6 and 7. This optimal colour vector is given by:

(1) $$i_{r}={\left[\Delta_{R}\;\;\;\Delta_{G}\;\;\;\Delta_{B}\right]}^{T}$$

where Δ*_R_*, Δ*_G_* and Δ*_B_* are the increments of the colour components *R*, *G* and *B*, respectively.

The optimal colour vector, **i_r_**, is injected in every frame of an image sequence in real-time applications to segment objects in colour images. Its efficiency has been especially tested in applications where a reduced contrast between the background colour and the colour of the object to segment exists, when there are illumination changes and the size of the object to segment is very small in comparison with the size of the captured scene.

An important property of the perceptual colour spaces (such as the *HSI* space) is that they produce a maximum disconnection between the chrominance and luminance components. As a result, the luminance can be almost fully isolated, making the segmentation process more invariant to the changes in shades and illumination as in [4]. For this reason, the analysis of the colour injection effects in the *HSI* space is made only using the *H* and *S* chromatic components (*HS* plane).

As the segmentation is performed using the *HS* components, we try to separate the representative vectors of the two classes (object and background) in angle (*H* component) and in magnitude (*S* component) using colour injection. However, special attention should be paid in this separation process to the variations of the dispersions (reliability) of both classes after the colour injection, because it has a very high incidence in the class separation process.

In short, if the original image is denoted by I, the optimal colour vector to add by **i_r_**, and the coloured image resulting of the colour injection by I_i_, is fulfilled:

(2) $$I_{i}=I+i_{r}$$

The algorithm proposed in this work is formed by two clearly different stages: an off-line and an on-line stage. The off-line stage is an initialization phase whose objective is to determine the optimal number of existing classes in the initial frame, and, from that, to obtain the object (O class) and background (B class) classes needed to carry out their separation. The off-line stage is explained in detail in Section 4. The result of this stage is the set of significant pixels (seeds) in the *RGB* space that represent both classes, identified by: O*_RGB_* = {**r_O1_**, **r_O2_**… **r_O*N*_**}, and B*_RGB_* = {**r_B1_**, **r_B2_**… **r_B*M*_**}, respectively, where **r**_**O***r*_ for *r* = 1, 2… *N* and **r**_**B***q*_ for *q* = 1, 2… *M* refer to the pixel vectors of the object and background classes, respectively.

**Figure 1.**
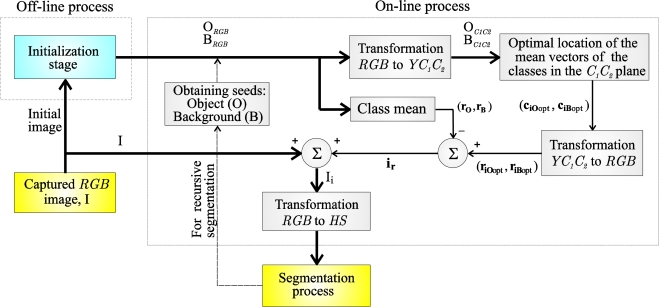
General block-diagram of the proposed algorithm to obtain the optimal colour vector (**i_r_**) to be injected to the captured image I. The off-line and on-line processes are grouped by discontinuous lines.

The on-line stage is the novel contribution of this paper. Its objective is to determine the optimal colour vector to inject (**i_r_**) for each frame in order to increase, optimally, the separation between classes O and B. The on-line process is executed before the segmentation process for each frame captured in real time. Figure 1 depicts the different phases of a segmentation process that uses the colour injection proposal of this paper.

The on-line process consists of the following stages:

1. For every O*_RGB_* and B*_RGB_* sample from the captured *RGB* image I, a transformation to the *YC_1_C_2_* space is done. Considering the chromatic components after the transformation, the resulting classes will be referred to as O*_C1C2_* = {**c_O1_**, **c_O2_**… **c**_**O***N*_} for the object class and as B*_C1C2_* = {**c_B1_**, **c_B2_**… **c**_**B***M*_} for the background, where the pixel vectors are denoted by “**c**”.
2. Using the properties of the *C_1_C_2_* plane and the relationship between the *HSI* and *YC_1_C_2_* colour spaces, the optimal location of the classes in the *C_1_C_2_* space is obtained by finding the optimal location of their respective mean vectors. The optimal location is the one that maximizes the class separation in the *HS* plane (maximum distance between the class means and minimum class dispersions). These optimal mean vectors will be referred to as **c**_**iO**opt_ and **c**_**iB**opt_. This phase is, undoubtedly, the most important of this work, and will be described in detail in subsequent sections.
3. From the mean vectors **c**_**iO**opt_ and **c**_**iB**opt_, their corresponding ones in the *RGB* space, **r**_**iO**opt_ and **r**_**iB**opt_, are calculated.
4. From the vectors **r_iOopt_** and **r**_**iB**opt_, and the mean vectors of the original classes (O*_RGB_*, B*_RGB_*) denoted by **r_O_** and **r_B_**, the optimal colour vector for injection is obtained. This optimal colour vector can be calculated from one of these expressions:
  (3) $$\begin{array}{ccccc} i_{r}=r_{\mathrm{iO}\text{opt}}-r_{O}, & & & & i_{r}=r_{\mathrm{iB}\text{opt}}-r_{B} \end{array}$$
5. Once the optimal colour vector has been obtained, the new “injected” image I_i_ can be calculated applying (2).

Finally, the coloured image I_i_ is transformed from the *RGB* space to the *HS* plane, where the segmentation is done, because the colour injection has its effects in the *HSI* space: the increase in the separation between the classes only happens in the *HSI* space or *HS* plane (in the *RGB* space the colour injection only produces a translation of the classes, keeping the distance between them constant, independently of the colour injection).

The proposed method can be implemented easily and can be used in real-time applications. In the following sections, the process to obtain the optimal vector for injection is presented in detail. At the end of this paper, in order to facilitate its reading, we have included three appendices with aspects related to the relationships between the *RGB*, the *HSI* and *YC_1_C_2_* spaces (Appendix A), statistical analysis of vectors in the *RGB* space and its relationships with the components in the *HSI* space (Appendix B), as well as the invariants of the mean vectors in the *C_1_C_2_* plane (Appendix C).

## 3. Criteria for the Separation between Classes

Since the objective of our work is to obtain a higher separation between the classes to facilitate the segmentation, it is necessary to define a measure of the efficiency of our proposal. The Fisher Ratio (*FR*) is frequently used to measure the efficiency in the class separability in classification systems [6,27,28]. This ratio quantifies simultaneously the inter-class separation and the internal dispersion (reliability) of the classes. For a two-class system, it is interesting to achieve a large distance metric between the class means and a minimum dispersion within each class (leading to a high *FR*). In this work, the *FR* is used as a pixel classification measurement index, using as discriminant features the *H* and *S* components of each pixel.

In a multi-class system, the generalized Fisher Ratio is expressed by [29]:

(4) $$\mathrm{FR}=\text{tr}\left(M_{w}^{-1}M_{b}\right)$$

where *M_b_* is the inter-class (between class) covariance matrix and *M_w_* is the internal (within class) dispersion matrix of the classes.

Equation (4) cannot be directly applied due to the circular form of the *H* component trajectory. There are two main reasons for this:

- For two-class systems (as our case is), *M_b_* may not represent the real angular distance between the hue means of the classes (the maximum angular distance between two vectors is π radians, even if one of the vectors is in the first quadrant of the *HS* plane and the other one in the fourth one). These problems have already been studied, for example in [8].
- The second reason is the discontinuity of the hue component when it moves from 2π to 0 radians (cyclic property). This implies that *M_w_* matrix does not represent the real hue variance of a class whose mean is close to 0 (2π). The reason is that some of the vectors would have small angles (close to 0), and some others would have very high ones (close to 2π), resulting in a wrong and high variance. The resulting *H* mean would also be wrong.

For the previous reasons, and supposing that the correlation between *H* and *S* is low, a particular *FR* has been defined. This *FR* is individually calculated for each component, and, as our space is bi-dimensional, is given by [29]:

(5) $$\mathrm{FR}={\mathrm{FR}}_{H}+{\mathrm{FR}}_{S}$$

where *FR_H_* and *FR_S_* represent the Fisher Ratio of the *H* and *S* components, respectively, and are given by:

(6) $$\begin{array}{ccc} {\mathrm{FR}}_{H}=\frac{\theta_{h}^{2}}{\sigma_{\mathrm{HO}}^{2}+\sigma_{\mathrm{HB}}^{2}}, & & {\mathrm{FR}}_{S}=\frac{{\left(S_{O}-S_{B}\right)}^{2}}{\sigma_{\mathrm{SO}}^{2}+\sigma_{\mathrm{SB}}^{2}} \end{array}$$

where *S_O_* − *S_B_* is the distance between the saturation means of both classes, *σ_SO_* and *σ_SB_* are the standard deviations of the saturation component for both classes, *θ_h_* is the separation angle between the hue means of both classes, and *σ_HO_* and *σ_HB_* are the standard deviations of the *H* component.

In (6) *θ_h_* ∈ [0, π] represents the real angular distance between the hue means, because θ_h_ = cos^−1^(CC_OB_). This avoids the aforementioned problem about the angular distance between the hue means of the mean vectors of both classes in the *HS* plane. *CC_OB_* is the correlation coefficient between the two mean vectors of the *RGB* components that have generated the mean vectors in the *HS* plane (Equation C.11) (see Appendix C). In this work, we have performed approximations in the calculation of *σ_HO_* and *σ_HB_* in order to avoid the problem of the hue discontinuity. Thus, the approximation for *σ_HO_* is: $\sigma_{\mathrm{HO}}^{2}=\sigma_{C\mathrm{Ho}}^{2}+\sigma_{S\mathrm{Ho}}^{2}$, where *σ*_C*Ho*_ is the standard deviation of every *cos*(*H_Or_*) for *r* = 1, 2… *N* and *σ*_S*Ho*_ is the standard deviation of every *sin*(*H_Or_*). *σ_HB_* is calculated with a similar method.

## 4. Initialization Stage (Off-Line Process)

**Figure 2.**
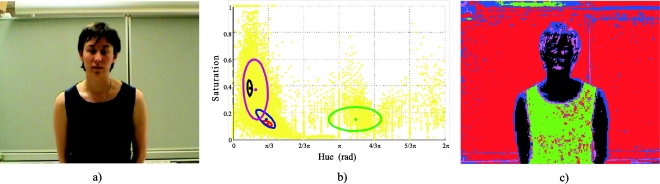
Class segmentation results of the initialization stage. (a) Initial image, (b) the K_opt_ Gaussians fitted to the classes projected in the *HS* plane, (c) segmented image corresponding with the K_opt_ classes in the Figure 2b. The colours of the different ellipses that represent the Gaussians in the Figure 2b correspond with the colours of the segmented regions in the image of the Figure 2c.

The first step of the off-line process is the capture of a first frame (initial image). The seed pixels that represent the classes O and B are obtained from this image, by means of any clustering technique used to identify the existing classes of the image, such as K-Means [30], GMM [13], *etc.* In this paper, the GMM technique is used (in the *HS* domain) because it provides highly reliable classes, and, as a final result, it also provides the mean vectors, the covariance matrixes and the a priori probabilities of the classes. The clustering by means of GMM uses the EM algorithm (Expectation Maximization) to obtain the optimal location and dispersion of a predefined image class number (K), projected in the *HS* plane. Therefore, a Gaussian model is assumed for each existing class, considering a uniform scene illumination. The GMM algorithm is applied several times, initializing it with different K values, in order to obtain the optimal number of existing classes in the image (K_opt_). K_opt_ corresponds with the K that produces the smallest error in the log-probability function of the EM algorithm, indicating that it is the best fit between the K Gaussians and the existing classes. Figure 2b shows the K_opt_ Gaussians projected in the *HS* plane, fitted to the existing classes in the initial image of the example in Figure 2a. Finally, Figure 2c depicts the original image segmentation as a function of the different existing classes (Figure 2b).

Once the GMM algorithm has converged, the following step is to find out the localization of the object class (O) in the HS plane. This off-line process is carried out easily, because the approximate location of the object class (O) in the *HS* plane is known at the beginning of the off-line process, as a result of the colour calibration adjustments of the camera. This approximate geometric locus in the *HS* plane is given by the mean vector **h_init_**. Taking that into account, the detection of the object class (O) is performed by simply selecting the class with the minimum Euclidean distance with **h_init._** We preferred the Euclidean distance over the Mahalanobis distance because the detection of the object class could be incorrect if **h_init_** is close to a class with high dispersion, and this class is also close to the object class (O). The reason of this effect is the consideration of the class covariance in the Mahalanobis distance.

Once the object class is detected, the next step is to select the background class (B). The background is usually formed by several classes, identified by {B_1_, B_2_… B_Kopt−1_}. Our objective is to select the B_k_ class that will be considered as representative of the background and that we will be identified simply by B. Among all the classes that form the background, we will select the B_k_ that:

(7) $$B=\text{arg}\;\underset{B_{k}}{\text{min}}\left(\frac{P_{\mathrm{FR}k}\left(B_{k}\right)}{P_{Bk}^{2}\left(B_{k}\right)}\right)$$

being *P_FR_*_k_; k = 1, 2… (K_opt_ − 1) the Fisher Ratio probabilities between the class O and each B_k_, defined by $P_{\mathrm{FR}k}=\left(\sqrt{{\mathrm{FR}}_{k}}/\sum_{k=1}^{\text{Kopt}}\sqrt{{\mathrm{FR}}_{k}}\right)$ where *FR*_k_ is the Fisher Ratio described in Section 3, and *P_B_*_k_; k = 1, 2… (K_opt_ − 1) the a priori probabilities of each B_k_ class to be the background class (B) of the image (given by the GMM algorithm).

Once the classes O and B have been identified, the seed pixels that represent both classes are obtained through an initial segmentation process of both the object class (O) and the background class (B). In this initial segmentation, the *pdf* (probability density function) of both classes in the *HS* plane are considered as unimodal bidimensional Gaussians, defined by the parameters obtained by the GMM clustering. This segmentation is carried out by selecting the pixels with higher probability to belong to the corresponding Gaussian. This stage of pixel selection is performed by truncating each class *pdf* with a determined threshold. This threshold corresponds to a percentage of the maximum probability of the corresponding bidimensional *pdf*, P_o_, for the class O, and P_b_ for the class B. The values of {P_o_, P_b_} ∈ [1, 0], have been experimentally set to P_o_ = 0.45 and P_b_ = 0.6, using the Receiver Operating Characteristic (ROC) curves obtained from the different tests performed with real images. In this case a ROC curve was obtained by each class (O and B), using a set of real images, without and with colour injection. The thresholds P_o_ and P_b_ correspond with the nearest values to the elbows of the ROC curves. As the pixel selection is carried out in the *HS* plane, it is necessary to truncate the *pdf* in the Intensity axis, in order to obtain pixel sets that reliably represent the classes O and B in the *RGB* space. The truncation of this unidimensional *pdf* is necessary because the *H* and *I* components are independent (Appendix A) (this is important when the clustering is carried out in the *HS* plane) and this generates correspondence problems when the pixels in *RGB* components are selected from its projections in the *HS* plane. In this second *pdf* truncation, the percentages selected of the maximum value of the *pdf* intensity of each class are P_fo_ for O, and P_fb_ for B. These percentages have been set to P_fo_ = 0.4 and P_fb_ = 0.5.

Once the previous process is completed, a random sampling is carried out, selecting *N* samples for the class O and *M* for the class B, in order to reduce the working space dimension. This is the method to obtain the sets O*_RGB_* and B*_RGB_* mentioned in Section 2.

## 5. Separation of the Classes in the *HS* Plane from Their Location in the *C_1_C_2_* Plane

This section details the most important relationships between the statistical mean and variance of the classes in the *C_1_C_2_* and *HS* planes. Also, the effect of adding the same vector (colour injection) to two vectors in *RGB* space on the projections of these vectors in the *C_1_C_2_* and *HS* planes is analyzed. This information is used to define an algorithm to easily calculate the optimal vector to inject in order to obtain the maximum separation between classes in the *HS* plane using translations in the plane *C_1_C_2_*.

### 5.1. Relationships between the HS and C_1_C_2_ Planes

Given two vectors in the *RGB* space, **r_O_** and **r_B_**, the resulting projection vectors in the *C_1_C_2_* plane, **c_O_** and **c_B_**, and in the *HS* plane, **h_O_** and **h_B_**, fulfil (see Appendix A):

(8) $$\theta_{c}=\theta_{h}=\theta$$

(9) $$\|c_{O}\|\neq \|h_{O}\|,\;\;\|c_{B}\|\neq \|h_{B}\|$$

(10) $${\|d_{c}\|}^{2}=g_{1}\left(c_{O},c_{B},\theta_{c}\right)={\|c_{O}\|}^{2}+{\|c_{B}\|}^{2}-2\|c_{O}\|\|c_{B}\|\text{cos}\theta_{c}$$

(11) $${\|d_{h}\|}^{2}=g_{2}\left(c_{O},c_{B},\theta_{h},I_{O},I_{B},f\left(H\right)\right)$$

where *θ_c_* is the angle between **c_O_** and **c_B_**, *θ_h_* the angle between **h_O_** and **h_B_**; **d_c_** is the distance vector between **c_O_** and **c_B_**, **d_h_** is the distance vector between **h_O_** and **h_B_**; and *I_O_*, *I_B_* are the intensity means of both classes, object and background, respectively, corresponding to the **h_O_** and **h_B_** vectors. *f*(*H*) is a weighting function that depends on the *H* component. *f*(*H*) ∈ [½, 1] (see Appendix B).

It is important to note that, since the *C_1_C_2_* plane is linear, when adding a vector **i_r_** (injected vector) to both **r_O_** and **r_B_** in the *RGB* space, the distance vector **d_c_** = **c_O_** − **c_B_** in the *C_1_C_2_* plane remains constant. These constant magnitude and orientation values (invariants of the **d_c_** vector) are denoted by ‖**d_c_**‖ and φ (see Appendix C). Therefore, colour injections in the *C_1_C_2_* plane result in class translations, as in the *RGB* space. This effect can be achieved with a translation vector **i_c_** (corresponding to **i_r_**) directly added in the *C_1_C_2_* plane.

Moreover, in the case of the *C_1_C_2_* plane, (10) is verified (cosine law). Therefore, given that ‖**d_c_**‖ remains constant for the different values of **i_r_**, the values of *θ*, ‖**c_O_**‖ and ‖**c_B_**‖, will be modified as a function of the value of **i_r_**. In the case of the *HS* plane, it must be said that if **i_r_** is added to the vectors **r_O_** and **r_B_** (contrary to what happens in the *C_1_C_2_* plane) the difference vector **d_h_** also varies. The reason is that, according to (11), **d_h_** depends on the value of *I_O_* and *I_B_* and on the *f*(*H*) weighting function. In any case (8) always holds.

In short, to calculate the value of the colour vector to be added in the *RBG* space to obtain a particular separation between the classes in the *HS* plane, the authors suggest using the relationships between the **h** vector components in the *HS* plane, and their corresponding **c** vector components in the *C_1_C_2_* plane, given by (Equation B.12) and (Equation B.13) (see Appendix B), and the relationship between pairs of vectors in these planes, given by (8, 9, 10 and 11).

Therefore, the proposed algorithm is based on the analysis of the behaviour of the vectors **c_O_** and **c_B_** in the *C_1_C_2_* plane and the properties of its difference vector **d_c_** (‖**d_c_**‖ and *φ* are invariant). These invariants allow us to establish a mathematical relationship between the class mean vectors before and after the colour injection. Thus, for example, the separation angle (hue difference) between two vectors in the *HS* plane can be easily controlled with the separation angle of the same vectors (**c_O_**, **c_B_**) in the *C_1_C_2_* plane, because both angles coincide (8).

**Figure 3.**
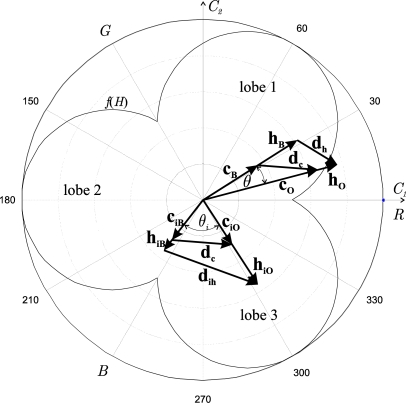
Correspondence between the mean vectors in the *C_1_C_2_* plane and the ones in the *HS* plane. The difference vector **d_c_** before and after the colour injection is shown.

In Figure 3, an example of the correspondence between the vectors **c_O_** and **c_B_** in the *C_1_C_2_* plane and the vectors **h_O_** and **h_B_** in the *HS* plane is shown. The relationships after performing the colour injection (vectors **c_iO_**, **c_iB_** and **h_iO_**, **h_iB_**) are also shown, as well as the difference vector **d_c_** before and after the colour injection, where the invariance in magnitude and angle can be observed. From now on, the “i” or “*i*” subscript refers to “colour injection”.

Figure 3 depicts how the translation of the vector **d_c_** has favoured the separation of the mean vectors of the classes in both components (*H* and *S*), because *θ_i_* > *θ*, and (‖**h_iO_**‖ − ‖**h_iB_**‖) > (‖**h_O_**‖ − ‖**h_B_**‖). An increase in the separation between the vectors after the colour injection can be verified (*θ_I_* > *θ*). However, the vector modules (saturation) decrease (‖**h_iO_**‖ < ‖**h_O_**‖, ‖**h_iB_**‖ < ‖**h_B_**‖), since there is an unavoidable compensation effect given by (10) (notice that for a fixed *I*, ‖**h**‖ = *const*‖**c**‖*f*(*H*)).

We could obtain a great number of class locations within the *HS* plane relocating **d_c_** with **i_c_** all over the *C_1_C_2_* plane. The determination of the optimal location is not a trivial task. In order to obtain an optimal **i_c_**, it is possible to apply learning techniques, such as fuzzy systems and neural networks, that take as parameters some functions derived from *FR*’s (6) and the invariants of the vector **d_c_**.

In the following Section (5.2), an algorithm for the calculation of the optimal **i_r_** (corresponding to optimal **i_c_**), conditioned to ‖**c_iO_**‖ = ‖**c_iB_**‖, is explained.

### 5.2. Separation between the Hue Means (Angular Separation)

The separation between the hue means is given by the angular separation between the vectors **h_iO_** and **h_iB_**, which indicate the colour separation. Once the expression of the distance between **h_iO_** and **h_iB_** is obtained, ‖**d_ih_**‖ (see Figure 3), an optimization process can be applied to it as a function of the *RGB* components of **i_r_** in order to obtain the optimal **i_r_** that produces the maximum separation needed. The problem when calculating the optimal colour vector is that it is not possible to obtain its analytical expression, mainly due to the discontinuities in the function of ‖**d_ih_**‖ (11).

In order to solve the problem posed by the discontinuities of ‖**d_ih_**‖ in the *HS* plane, the authors propose to use the *C_1_C_2_* plane, where the distance function between the vectors **c_iO_** and **c_iB_**, (‖**d_c_**‖) (10) does not present discontinuities, and, as well, remains constant in magnitude and direction for different injections of colour vectors.

The interrelationship (due to the invariants of the vector **d_c_** and the relationships between the *HS* and *C_1_C_2_* planes) between the angle (*θ_i_*) that the vectors **c_iO_** and **c_iB_** form and their modules (10) should be taken into account to obtain the separation angle (hue difference) of two vectors in the *HS* plane. Therefore, the maximum separation angle between the vectors may imply (due to the compensation effect) a diminution of their modules, and, consequently, the saturation of both vectors. The saturation reduction of the vectors **h_iO_** and **h_iB_** implies that they become closer to the achromatic zone (the origin of the coordinates system), which means that the colours approximate to gray scale. The consequence of this phenomenon is the loss of discriminating power in the segmentation.

Therefore, the proposed algorithm has been parameterized as a function of the mentioned separation angle *θ_i_* between the vectors **c_iO_** and **c_iB._** In our case, the optimal angle *θ_i_* is obtained from an observation function that measures the effectiveness of the class separation in different locations in the *HS* plane. This function will be described in paragraph *f* of Section 6.

**Figure 4.**
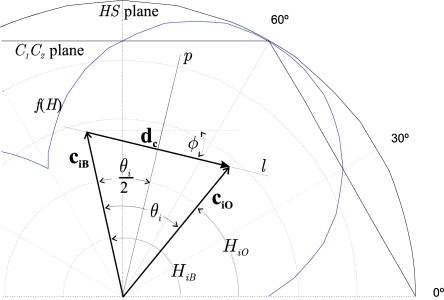
Location of the vectors **c_iO_** and **c_iB_** in the *C_1_C_2_* plane once the colour injection has been performed.

When the angle of separation *θ_i_* reaches a maximum, *θ_i_* coincides with the angle whose bisector is a straight line *p*, which passes through the origin of coordinates and is perpendicular to the straight line, *l*, whose director vector is **d_c_** (Figure 4).

Therefore, the vector for injection (**i_r_**) that causes the maximum hue difference, causes the modules of both vectors **c_iO_** and **c_iB_** to become equal (‖**c_iO_**‖ = ‖**c_iB_**‖). It also causes the distance between the intersection point of the lines *p* and *l* and the extreme of each vector to be ‖**d_c_**‖/2. Figure 4 illustrates an example of the location of the vectors **c_O_** and **c_B_** after the injection of the colour vector (**c_iO_** and **c_iB_**) with those imposed restrictions.

The authors have given more importance to the angular (*H*) separation, because increasing both *H* and *S* at the same time is not possible. The main reason is that *H* has a discrimination power higher than *S*. Besides, the *H* component is totally uncorrelated to the *I* component, which does not occur to the *S* component (see Appendix B). Parameterization only by *θ_i_* implies that we can only control the separation between the hue means. The starting point to obtain the distance between the saturation means is mainly the location of the vectors **c_iO_** and **c_iB_** with respect to the saturation weighting function in the *C_1_C_2_* plane. As can be observed, in this process there is no control on the *S* component, so its contribution on the class separation will depend on the modification of the statistics of this component with the variation of *θ_i_*.

### 5.3. Separation between the Saturation Means (Saturation Difference)

In this section, an analysis of the behaviour of the separation between the saturation components of two vectors in the *HS* plane is performed. Given two vectors, for example **h_iO_** and **h_iB_**, in the *HS* plane, we analyze how the value of the saturation difference between both vectors *S_O_* − *S_B_* = ‖**h_iO_**‖ − ‖**h_iB_**‖ varies. In our case, as ‖**c_iO_**‖ = ‖**c_iB_**‖ = *C_i_*, then the intensities (*I_O_*, *I_B_*) corresponding to both vectors **h_iO_** and **h_iB_**, and the value of the saturation weighting function *f*(*H*) of each one, are the parameters with significant effect in the value of *S_O_* − *S_B_*. The reason is that, according to (Equation B.11), the difference *S_O_* − *S_B_* will only have a non-zero value if *I* and *f*(*H*) of both vectors are different (notice that the saturation varies inversely with the intensity, and directly with *f*(*H*)). As an example, Figure 3 shows vectors **h_iO_** and **h_iB_** (overlapped to their respective vectors **c_iO_** and **c_iB_**), as well as the saturation weighting curve *f*(*H*). In the case of Figure 3, the colour injection is done supposing *I_O_* = *I_B_*, therefore, the weighting function *f*(*H*) is the only responsible for the difference in the module of the vectors **h_iO_** and **h_iB_**, that is, of the separation between the saturation means of both classes. As previously indicated, in our proposal there is no control of the *S_O_* − *S_B_* value, but its behaviour as a function of the colour injections performed, parameterized by *θ_i_*, is known. According to this, it can be said that *S_O_* − *S_B_* is determined, as expressed in (12), by: (*a*) the intensities of the vectors **h_iO_** and **h_iB_** (*I_O_*, *I_B_*), and (*b*) the module and angle of **d_c_** (the invariants) since these determine the location of the vectors **h_iO_** and **h_iB_** along the curve *f*(*H*) in the *HS* plane. In the case of Figure 3, where **h_iO_** is located in the third lobe and **h_iB_** in the second, it is fulfilled:

(12) $$S_{O}-S_{B}=k_{1}\text{cot}\left(\theta_{i}/2\right)+k_{2}$$

where:

$$\begin{array}{l} k_{1}=\left\|d_{c}\right\|\left(I_{B}\;\text{cos}\left(5\pi /6+\varphi \right)+I_{O}\;\text{cos}\left(\pi /2+\varphi \right)\right)/3I_{O}I_{B}\;\text{and}\\ \;\;k_{2}=\left\|d_{c}\right\|\left(I_{O}\;\text{sin}\left(\pi /2+\varphi \right)-I_{B}\;\text{sin}\left(5\pi /6+\varphi \right)\right)/3I_{O}I_{B}. \end{array}$$

### 5.4. Analysis of the Class Dispersion

In order to obtain the optimal vector for injection, **i_r_**, by means of the suitable election of *θ_i_*, we should take into account not only the information given by the mean vectors **c_O_** and **c_B_** in the *C_1_C_2_* plane, but also the dispersion of the distributions of both classes.

In this section we analyze the behaviour of the class dispersions in the *HS* plane, that is, how the hue and saturation dispersions are affected when the classes are translated in the *C_1_C_2_* plane, as a result of the colour injection. A class separation measurement function will be defined to quantify the effectiveness of the colour injection. This analysis will be necessary to understand how the *H* and *S* dispersions are modified with the colour injection, in addition to the performance of the class separation measurement function.

#### 5.4.1. Hue dispersion (Angular dispersion)

The hue dispersion is determined by the effects of the dispersion transformation when passing from the *C_1_C_2_* plane to the *HS* plane. If **R_o_** is the (2 × *N*) matrix formed by the *N* vectors of the O class: **c**_**O***r*_; *r* = 1, 2… *N*, before any translation, the parameters of the O class uncertainty ellipse, *i.e.*, the hue dispersion invariants, are obtained from the covariance matrix of **R_o_**, by:

(13) $$\omega_{O}={\text{tan}}^{-1}\left(C_{2\mathrm{Ou}}/C_{1\mathrm{Ou}}\right)$$

where *ω_O_* is the angle formed by the semi-major axis of the class uncertainty ellipse with respect to the horizontal axis (*C_1_*), and *C_1Ou_* and *C_2Ou_* are the eigenvector components corresponding to the highest eigenvalue (*λ_Ou_*) of the covariance matrix. The semi-major and semi-minor uncertainty ellipse axes, *u_O_* and *l_O_* respectively, which represent the maximum and minimum variance, are given by:

(14) $$u_{O}=\sqrt{\lambda_{\mathrm{Ou}}},\;l_{O}=\sqrt{\lambda_{\mathrm{Ol}}}$$

where *λ_Ol_* is the minor eigenvalue of the covariance matrix. From these dispersion invariants, it is possible to obtain the model for the hue dispersion. Therefore, our interest is to obtain a correspondence between the hue dispersions in the *HS* plane by means of the information offered by the angular dispersion in the *C_1_C_2_* plane. Knowing that the variation of the angular dispersion in the *C_1_C_2_* plane corresponds with the variation of the hue dispersion in the *HS* plane, and since the *C_1_C_2_* plane is a Cartesian plane, the problem is posed in the polar coordinates, taking these two considerations into account:

- As previously indicated, in the *C_1_C_2_* plane, the colour injections only produce translations of the classes and, therefore, variations of their mean vector modules (‖**c_iO_**‖, ‖**c_iB_**‖). This causes the modification of the angular dispersions of both classes, because they depend on *C_i_* = ‖**c_iO_**‖ = ‖**c_iB_**‖ (distance between the dispersion centre and the origin of the *C_1_C_2_* plane). These effects of the hue dispersion modification have been observed when performing translations of a class by adding Gaussian noise in the *RGB* space [22,31]. In conclusion, the angular dispersion increases when the magnitude of its respective mean vector decreases due to the increment of the separation angle *θ_i_*, according to:
  (15) $$C_{i}=\|d_{c}\|/2\text{sin}\left(\theta_{i}/2\right)$$
- The geometric forms of the class distributions are not predetermined, but they can vary since they depend on the samples randomly taken from the object and the background. The colour injections produce class translations in the *C_1_C_2_* plane, implying that from the point of view of the *HS* plane, the dispersion also depends on the geometric form of the classes. The reason is that, for different translations of a class, different orientations between the axis of maximum and minimum dispersion (represented by their uncertainty ellipse in a *C_1_C_2_* plane) with respect to the orientation of their mean vectors (**c_iO_** or **c_iB_**) are generated. Therefore, independently of the class mean vector module, a distance *d_a_* exists that contributes to the angular deviation. This distance *d_a_* depends only on the geometric form and orientation of the dispersion after each translation. Then, *d_a_*, in this case for the O class, will depend on the values of *ω_O_*, *u_O_* and *l_O_* given by (13) and (14). This *d_a_* can be approximated by means of the distance between the centre of the uncertainty ellipse and the intersection point between two right lines: one is the tangent line to the ellipse which at the same time passes through the origin of plane, and the other line is perpendicular to the previous one and it crosses the centre of the ellipse. With *d_a_* and (15) the angular deviation can be approximated by:
  (16) $$\sigma_{\mathrm{iH}}={\text{sin}}^{-1}\left(d_{a}/C_{i}\right)$$

**Figure 5.**
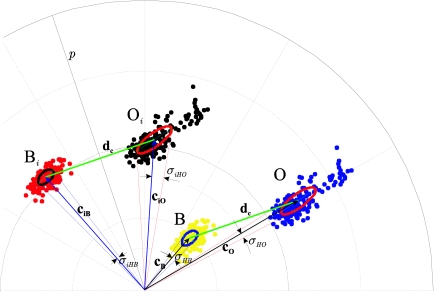
Uncertainty ellipses of the classes O and B in the *C_1_C_2_* plane: before the colour injection: O (blue) and B (yellow) and after the colour injection: O (black) and B (red). Geometric approximation of the hue deviations of the classes, as a function of the ellipse locations. The different alignments of the axes of the ellipse with respect to the direction of the mean vectors of each class are shown.

As an example, in Figure 5 we depict the object class (O) before a translation, for the addition of a vector **i_c_** in this *C_1_C_2_* plane, or, for the injection of a vector **i_r_**, directly to the classes in *RGB* components. Over the object class, its respective uncertainty ellipse is shown.

In Figure 5, we can observe that the semi-major axis of the ellipse is relatively aligned to the mean vector **c_O_** of the class, causing the perception of the minimum angular dispersion of that class. It can also be observed that the module of this mean vector, **c_O_**, before the injection is greater than the module of the vector after it has been injected, **c_iO_**, which, therefore, is also perceived as a minor angular dispersion by this effect. We may conclude then, that the initial location of this class in the *HS* plane represents a very favourable case, since the angular deviation before the colour injection is small.

Nevertheless, for the background class (B) before the colour injection, certain alignment between the mean vector **c_B_** and the axis of greater dispersion of this class can also be observed, implying a reduced angular deviation. However, the problem is that the module of the vector **c_B_** is reduced and, therefore, the angular deviation increases. In this case, it can be observed in Figure 5 that after the colour injection, the angular dispersion of the class B*_i_* is smaller, since the module **c_iB_** is greater.

**Figure 6.**
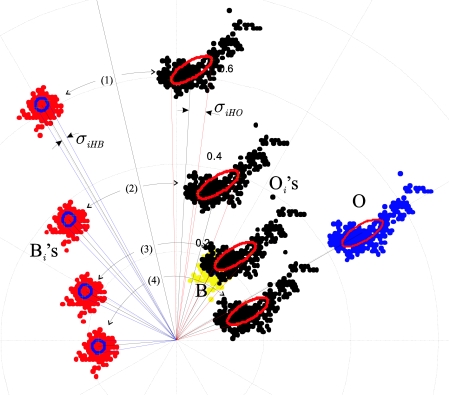
Location of the classes for 4 different separation angles (*θ_i_*) in the polar *HS* plane: (1) *θ_i_* = 33°, (2) *θ_i_* = 57°, (3) *θ_i_* = 97°, (4) *θ_i_* = 163°. The original classes (O and B) and the injected classes are shown for the 4 colour injections (O*_i_*′s and B*_i_*′s).

Figure 6 depicts another example, with the different class locations after four colour injections. The modifications of the angular deviations *σ_iHO_* and *σ_iHB_* of the object and background classes as a function of the orientation of their respective uncertainty ellipses and the modules of their respective mean vectors can be observed.

#### 5.4.2. Saturation dispersion

The dispersion of the saturation component is not directly affected by the class translations (due to the colour injections) in the *C_1_C_2_* plane, if all the class vectors have the same intensity. The reason is that the saturation is a linear function of the *C_1_* and *C_2_* components. The expression of the saturation for lobe 1 of *f*(*H*) is (Equation B.13) (see Appendix B):

(17) $$S=\frac{C_{1}}{3I}+\frac{C_{2}}{\sqrt{3}I}$$

This characteristic of linearity in the *C_1_C_2_* plane makes the deviation of the saturation (*σ_S_*) constant, since the distance between vectors in the *C_1_C_2_* plane remains constant, independent of the colour injection. Nevertheless, in the *HS* plane *σ_S_* will be different for each lobe of *f*(*H*) but will stay constant within each lobe. Evidently, if the class vectors have different intensity, the dispersion of the saturation will not be constant for each location, not even within the lobes (there is a greater variation of *σ_S_* when the dispersion of the intensity component is greater).

**Figure 7.**
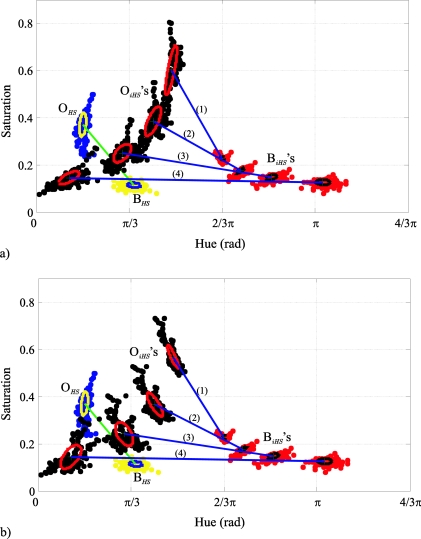
Classes projected in an *HS* Cartesian plane, corresponding with the example of the Figure 6, where the dispersion variation of both classes with the colour injections is observed. (a) A variable *S* deviation is shown because the classes keep their original intensities: *σ_IO_* ≠ *σ_IB_* ≠ 0, (b) A constant *S* deviation is shown because each class intensity is equal to its respective intensity mean, *i.e.*, *σ_IO_* = *σ_IB_* = 0.

Figure 7 illustrates how the hue and saturation dispersions are modified for the four colour injections of Figure 6.

In this case, the locations of both classes are projected in an *HS* Cartesian plane. The magnitude of the *H* and *S* deviation can be appreciated by means of the projections of the corresponding uncertainty ellipses of both classes in the axes *H* and *S.* In Figure 7a we can observe a diminution of the *H* deviation and the increase of the *S* deviation when the angle *θ_i_* between the classes decreases, because the modules of the mean vectors of both classes increase. It can also be observed how the *S* deviation of the O*_i_* class, is modified more than the deviation of B*_i_*, because the *I* dispersion of O*_i_* is greater. Figure 7b shows the same example as Figure 7a, but with the intensities of the class vectors equal to its intensity mean, *i.e.*, *I*_*O*1_ = *I*_*O*2_ = ... = *I_ON_* = *I_O_*, and *I*_*B*1_ = *I*_*B*2_ = … = *I_BM_* = *I_B_*, implying that *σ_IO_* = *σ_IB_* = 0. Then, we can see how the *S* deviation of O*_i_*, remains constant for each colour injection.

However, our interest in this paragraph is to understand how the colour injections affect the saturation dispersion. This is the reason why in our algorithm the *S* deviations of both classes are obtained considering their original intensities.

## 6. Algorithm for the Optimal Location of the Mean Vectors of Both Classes in *C_1_C_2_* Plane

This section presents the strategy used to obtain, in the *C_1_C_2_* plane, the mean vectors that maximize the separation between the classes in the *HS* plane. This section constitutes the main stage in Figure 1: “Optimal location of the mean vectors of the classes in the *C_1_C_2_* plane”. As shown in Figure 1, for each captured image, an algorithm to obtain the optimal location in the *C_1_C_2_* plane of the mean vectors of both classes (object and background) is executed. From these optimal vectors, **c**_**iO**opt_ and **c**_**iB**opt_, and once the transformation to the *RGB* space is performed (**r**_**iO**opt_, **r**_**iB**opt_), the optimal vector to inject, **i_r_**, is obtained using (3).

**Figure 8.**
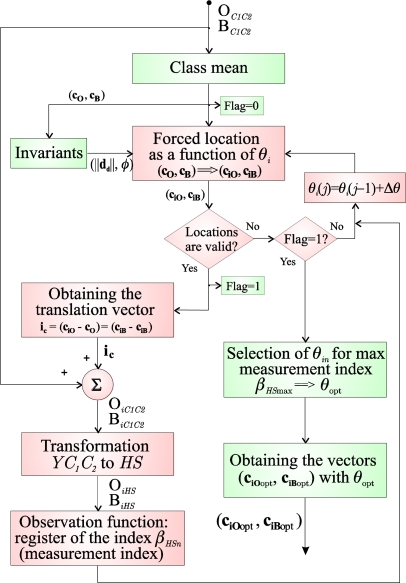
Functional diagram to obtain the optimal location of the mean vectors in the *C_1_C_2_* plane: **c_iO_**_opt_ and **c_iB_**_opt_.

The proposal to obtain these optimal vectors, **c**_**iO**opt_ and **c**_**iB**opt_, consists of different phases, and its general block diagram is depicted in Figure 8.

As can be observed, the proposal includes an iterative algorithm to obtain a set of locations for the mean vectors of the classes (**c_iO_** and **c_iB_**) in the *C_1_C_2_* plane. The location of each vector will be parameterized by the angle formed between both vectors, *θ_i_*. Therefore, we try to obtain a set of *θ_in_* (*θ*_*i*1_, *θ*_*i*2_…). Each of them will have associated a measurement index of separation between classes that we will identify by *β_HSn_* (*β*_*HS*1_, *β*_*HS*2_ …). From the function *β_HSn_* = *f* (*θ_in_*), the value of *θ_in_* that produces the maximum separation between classes is obtained, *θ_in_* optimal: *θ*_opt_.

The process begins obtaining the mean vectors of each class in *C_1_C_2_* plane. These mean vectors will be,

(18) $$\begin{array}{llll} c_{O}=E\left\{c_{O1},c_{O2}\ldots c_{ON}\right\}, & & & c_{B}=E\left\{c_{B1},c_{B2}\ldots c_{BM}\right\} \end{array}$$

From the vectors **c_O_** and **c_B_**, its difference vector, **d_c,_** is obtained. As previously indicated, the magnitude, ‖**d_c_**‖, and angle, *φ*, of the vector **d_c_** are invariant against translations in the *C_1_C_2_* plane. Their values are given by equation (19):

(19) $$\begin{array}{ccc} \left\|d_{c}\right\|={\left(d_{C1}^{2}+d_{C2}^{2}\right)}^{1/2}, & & \varphi =\{\begin{array}{l} {\text{cos}}^{-1}\left(d_{C1}/\left\|d_{c}\right\|\right);d_{C2}\geq 0\\ 2\pi -{\text{cos}}^{-1}\left(d_{C1}/\left\|d_{c}\right\|\right);d_{C2}<0 \end{array} \end{array}$$

where *d_C1_* = *C_1O_* − *C_1B_* and *d_C2_* = *C_2O_* − *C_2B_*, such that (*C_1O_*, *C_2O_*) and (*C_1B_*, *C_2B_*) are the components of the vectors **c_O_** and **c_B_**, respectively.

The iterative process consists of the following six steps:

(a) Forced location of the mean vectors in the C_1_C_2_ plane

The original vectors **c_O_** and **c_B_** are relocated (forced) in the *C_1_C_2_* plane using the invariants (‖**d_c_**‖, *φ*), obtaining the new vectors (**c_Io_** and **c_Ib_**). Each location of the vectors (**c_Io_** and **c_Ib_**) should fulfil the following geometric restriction: the straight line that passes through the origin of the *C_1_C_2_* plane and is perpendicular to the vector **d_c_** should intersect this last one in ‖**d_c_**‖/2. As previously indicated, this implies that:

(20) $$C_{i}\;=\;\left\|c_{\mathrm{iO}}\right\|\;=\;\left\|c_{\mathrm{iB}}\right\|=\left\|d_{c}\right\|/\left(2\text{sin}\left(\theta_{i}/2\right)\right)$$

This *θ_I_* is the parameter to vary in order to obtain the different locations of the vectors **C_IO_** and **C_IB_**, and, therefore, of the locations of the classes in the *C_1_C_2_* plane.

The Cartesian components of these vectors (Figure 4), particularized for the vector **C_IO_**, are given by:

(21) $$\begin{array}{ccc} C_{1\mathrm{iO}}=C_{i}\text{cos}\left(H_{\mathrm{iO}}\right), & & C_{2\mathrm{iO}}=C_{i}\text{sin}\left(H_{\mathrm{iO}}\right) \end{array}$$

where *h_IO_* is the angle of the vector that can be expressed by:

(22) $$H_{\mathrm{iO}}=\pi /2+\varphi -\theta_{i}/2$$

Similar expressions can be obtained for **C_IB_**.

The iterative algorithm is initialized with an *θ_i_* equal to *θ* (*θ* is the angle formed by the vectors **c_O_** and **c_B_)**. In each iteration (*j*) of the algorithm, the value of *θ_i_* is increased: *θ_i_*(*j*) *= θ_i_*(*j* − 1) *+ Δθ*.

We should also take into account that *θ_i_* represents the hue distance between the mean vectors (**H_IO_** and **H_IB_**) of the classes in the *HS* plane. This indicates that a direct relationship exists between the class translations in the *C_1_C_2_* plane and the hue separation distance between the class means in the *HS* plane.

(b) Verification of the validity for the locations of the **C_IO_** and **C_IB_** vectors

For each increase of *θ_i_*, the validity of the locations of the vectors **C_IO_** and **C_IB_** is verified. In case they are valid, the value of *θ_i_* is included in the set *θ_in_*. The validity of **C_IO_** and **C_IB_** (validity of *θ_i_*) is tested by checking if the components of the corresponding vectors in *RGB* space (**R_IO_**, **R_IB_**) fulfil the limitations imposed by this space, *i.e.*, the values are in the range [0, 1], because they are normalized with respect to 255.

(c) Calculation of the class translation vector in the C_1_C_2_ plane

The translation vector **i_c_** is obtained for each value of *θ_in_*. This vector **i_c_** is responsible for the class translations from its original position to the forced location defined by *θ_in_*. The translation vector **i_c_** in the *C_1_C_2_* plane corresponds to the vector to inject **i_r_** in the *RGB* space. This translation vector can be calculated from any of the following expressions:

(23) $$i_{c}=c_{\mathrm{iO}}-c_{O},\;\;i_{c}=c_{\mathrm{iB}}-c_{B}$$

(d) Translation of the classes in the C_1_C_2_ plane

The class translations in the *C_1_C_2_* plane are performed with the value of **i_c_** that has been calculated. Therefore, each vector **c** belonging to the object and background classes are increased by **i_c_**:

(24) $$\begin{array}{cccccc} O_{\mathrm{iC}1C2}=\left\{c_{Or}+i_{c}\right\}; & r=1,2\ldots N, & & & B_{\mathrm{iC}1C2}=\left\{c_{Bq}+i_{c}\right\}; & q=1,2\ldots M \end{array}$$

(e) Class transformation from the C_1_C_2_ plane to the HS

The classes in the *HS* plane (o*_IhS_* and b*_IhS_*) are obtained from the translated classes o*_IC1C2_* and b*_IC1C2_*, using (Equation B.12), (Equation B.13) and (Equation B.14).

(f) Observation function: calculation of the class separation measurement index (*β_HSn_*) in the *HS* plane

As the class separation observation function, a normalized measurement index has been defined (*β_HS_*) from the *FR* described in (5). It has been normalized to obtain *β_HSn_* = 1 when the class separation is maximum. To obtain the *β_HSn_* corresponding with each *θ_in_*, we consider the mean and the dispersion of *H* and *S* of the classes, according to (6). Therefore, two class separation measurement indexes as a function of *θ_in_* have been defined, one for each component:

(25) $$\begin{array}{cccccc} \beta_{\mathrm{Hn}}=\left(\sqrt{{\mathrm{FR}}_{H}}-1\right)/\sqrt{{\mathrm{FR}}_{H}}, & & & & & \beta_{\mathrm{Sn}}=\left(\sqrt{{\mathrm{FR}}_{S}}-1\right)/\sqrt{{\mathrm{FR}}_{S}} \end{array}$$

The final class separation measurement index is given by:

(26) $$\beta_{\mathrm{HSn}}=k_{h}\;\beta_{\mathrm{Hn}}+\left(1-k_{h}\;\right)\beta_{\mathrm{Sn}}$$

where *k_h_* is a weighting factor between *β_Hn_* and *β_Sn_*. The value of *k_h_* ∈ [0, 1] is chosen depending on the prominence we want to give *H* or *S* in the segmentation process. Taking into account that *H* has a greater discriminating power than *S*, *k_h_* > ½ should be fixed.

This iterative process is repeated until the first non valid value of *θ_in_* is generated, and the pairs (*β_HSn_*, *θ_in_*) are registered to obtain the function *β_HSn_* = *f*(*θ_in_*) afterwards.

Once the set of pairs (*β_HSn_*, *θ_in_*) is obtained, the *θ_in_* that produces the maximum class separation measurement index is selected. A cubic interpolation is performed around that local maximum to obtain the maximum of the interpolation index, *β_HS_*_max_, and its associated angle, *θ*_opt_. Finally, with this *θ*_opt_, the **c_iO_**_opt_ and **c_iB_**_opt_ vectors are obtained using (20), (21) and (22).

**Figure 9.**
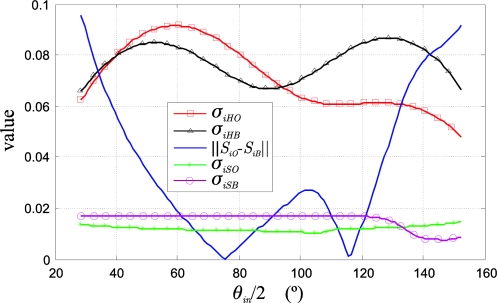
Hue and saturation deviation of both classes as a function of *θ_in_*/2. The difference between the saturation means of both classes is shown too.

As an example, Figure 9 shows the variation curves, as a function of *θ_in_*/2, of the statistical data: deviations of hue (*σ_iHO_* and *σ_iHB_*), deviation of saturation (*σ_iSO_* and *σ_iSB_*), and difference between the saturation means ‖*S_iO_* − *S_iB_*‖ needed to obtain the different class separation measurement indexes (25).

## 7. Calculation of the Optimal Colour Vector to Add and the Effects that it Produces on the Images

The calculation of the optimal colour vector to add, **i_r_**, is the goal of our proposal, because this vector changes the colours of the captured image in a suitable manner, so that the classes separate and, therefore, the object class can be more easily segmented.

**Figure 10.**
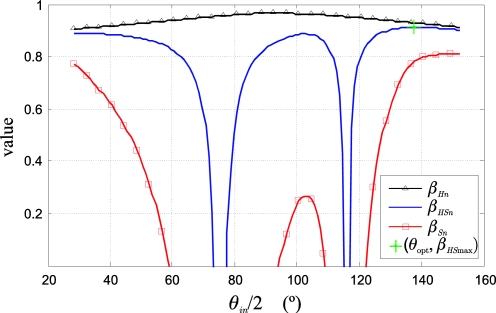
Measurement indexes as a function of *θ_in_*/2.

Figure 10 shows the values of *β_Hn_*, *β_Sn_* and *β_HSn_* obtained from the values of the statistical data depicted in Figure 9. The values of (*θ*_opt_, *β_HS_*_max_) obtained by interpolation are also shown.

As depicted in the block diagram of Figure 1, once the vectors **c**_**iO**opt_ and **c**_**iB**opt_, that represent the optimal location of the classes in the *HS* plane, are obtained, the vectors **r**_**iO**opt_ and **r**_**iB**opt_ can be calculated. Thus, for instance, for the object class, O: if *C*_*1O*opt_ and *C*_*2O*opt_ are the *C_1_* and *C_2_* components of the vector **c**_**iO**opt_ respectively, the vector **r**_**iO**opt_ in *RGB* space is obtained by:

(27) $$r_{\mathrm{iO}\text{opt}}=Q^{-1}{\left[Y_{\mathrm{iO}}\;\;\;C_{1O\text{opt}}\;\;\;C_{2O\text{opt}}\right]}^{T}$$

where **Q** is the transformation matrix (Equation A.2) and *Y_iO_* is the intensity mean of the object class translated in the *C_1_C_2_* plane. The **i_r_** vector is obtained with this **r_iO_**_opt_ applying (3). Considering that the colour injection can be made without modifying the mean intensity of the class after the injection of **i_r_**, *Y_iO_* = *I_O_* holds. Although it is possible to modify the saturation mean varying the intensity mean, in this case, we want the saturation mean to be only affected by the *f*(*H*) value and the Chroma component (*C*). Therefore, the vector to inject, **i_r_**, should have zero mean (E{**i_r_**} = 0). The fact that E{**i_r_**} = 0 implies that the intensity mean of the original image (I) and the injected one (I_i_) are equal.

**Figure 11.**
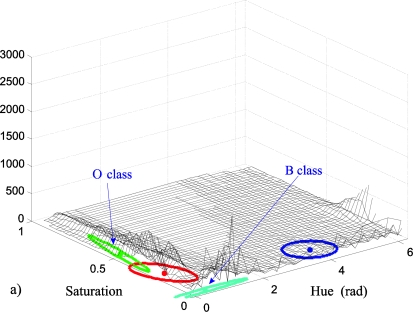

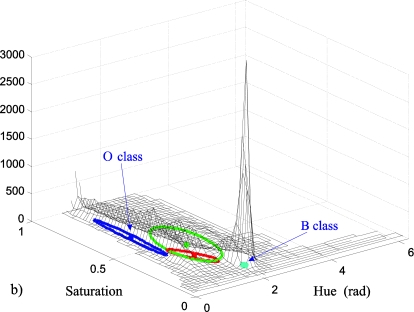
Example of 2D histograms in the *HS* plane for the original image I and injected image I_i_. (a) Original image histogram, (b) injected image histogram. The class redistribution in the injected image when compared with the original image can be observed. In the image I_i_, an isolation of the main scene classes (O and B) can be visually appreciated, as well as a shape closer to the Gaussian form.

**Figure 12.**
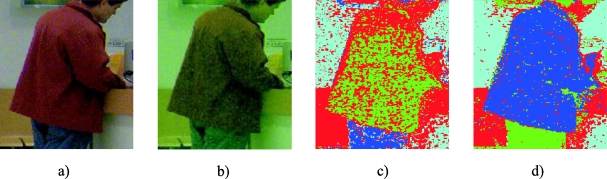
Reference images for the example of Figure 11. Example of original and injected images segmentations: (a) original image, I, (b) injected image, I_i_, (c) original image segmentation according to the projected classes in Figure 11a and (d) injected image segmentation according to the projected classes in Figure 11b.

The effect of injecting the vector **i_r_** to the original image in the new image, I_i_, is a greater concentration of the pixel colours around the mean colour of each one of the two classes. That is, the colour injection contributes to the histogram equalization of the captured image in the *HS* plane. This equalization has a concentration effect on each class, and, therefore, the injection of **i_r_** contributes to approaching the class distributions to a Gaussian shape. As an example, Figure 11 shows the 2D histograms of image I (Figure 11a) and of the coloured image resulting from the colour injection I_i_, (Figure 11b), for a particular case (Figure 12 images).

In these figures (11a and 11b), the equalization of the histogram produced by the effect of the colour injection can be clearly observed. The segmentation of both images is shown in Figure 12c and 12d respectively. In this example, K_opt_ = 4, the O class corresponds with the jacket and the B class with the wall.

**Figure 13.**
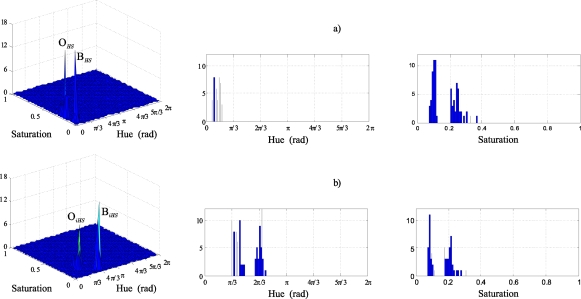
Histograms of the O*_HS_* and B*_HS_* sets: (a) before the colour injection, (b) after the colour injection.

The effect of the class separation between O and B classes can also be directly seen, analyzing the class locations before and after the colour injection in their histograms. Figure 13 shows the histograms corresponding to the sets O*_HS_* and B*_HS_* in (a), and the sets O*_iHS_* and B*_iHS_* in (b). A remarkable increase in the hue component separation can be observed in the histograms of Figure 13b due to the colour injection.

The rest of the image classes different from B, B*_x_* ≠ B; *x* = 1, 2 … K_opt_ − 2, are also affected by the effects of the colour injection. In this sense, as the class selected as B is the closest to the class O that also has a high probability to be the image background (fulfils equation (7)), when the separation between the classes O and B increases, the classes B*_x_* also increase their separation with the class O. However, the colour injection decreases the separation between the class O and those classes B*_x_* (B′*x; x* = 1, 2 …) that are closer than B to the class O but that were not selected as class B because they had a lower a priori probability. The consequence is that these classes (B′*x*) can be considered as class O, producing false positives in the object pixel classification.

Another effect of the colour injection is the automatic compensation of the illumination changes. That is, due to the equalization and the separation of the classes O and B in the injected image, there is a minimization of the problems produced by the illumination changes. The reason is that the main colour component affected by the illumination changes is *S*, and, as previously explained, our algorithm gives more importance to the separation of the most discriminant component, *H*. Then, both classes, O and B, always keep a certain separation, independently of the parameter variation of both distributions, and mainly when the mean and variance of the *S* component vary due to changes in the luminous intensity.

**Figure 14.**
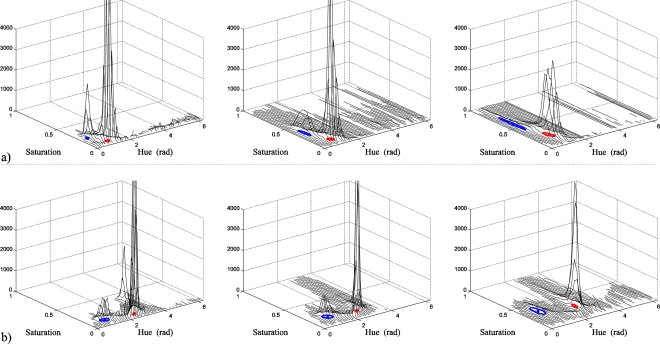
Three 2D Histograms for three intensity mean values for each image, I and I_i_: *I_m_*_1_ = 0.70, *I_m_*_2_ = 0.45 and *I_m_*_3_ = 0.21. (a) histograms of the image I, (b) histograms of the image I_i_. We can observe how the distribution statistics of both classes of the image I_i_ are less affected by the illumination changes than the ones of image I.

Next, in Figure 14, three histograms are presented, for the original and the injected image. All of them have been obtained with the different mean luminous intensity of the image (*I_m_* = E{I} = E{I_i_}): (*I_m_*_1_ = 0.70, *I_m_*_2_ = 0.45 and *I_m_*_3_ = 0.21). The illumination compensation effects mentioned above can be observed in this figure.

## 8. Experimental Results

A bank of real images from different scenes has been used in a first phase of the practical tests, in order to evaluate the effectiveness of the proposed method. Here, a Gaussian classifier has been used as a segmentation technique, supposing a unimodal Gaussian model for the respective object and background class-conditional *pdfs*, *i.e.*, *p*(**h_i_**|O_i_) and *p*(**h_i_**|B_i_). Thus, *p*(**h_i_**|O_i_) = *g*(**h_i_**; **h_iO_**, **Σ_iO_**) is given by:

(28) $$p\left(h_{i}|O_{i}\right)=\frac{1}{2\pi {\left|\Sigma_{\mathrm{iO}}\right|}^{1/2}}\text{exp}\left\{-\frac{1}{2}d_{m}\right\};\;\;d_{m}={\left(h_{i}-h_{\mathrm{iO}}\right)}^{T}\Sigma_{\mathrm{iO}}^{-1}\left(h_{i}-h_{\mathrm{iO}}\right)$$

where **h_i_** represents each pixel of the image I_i_, and **Σ_iO_** is the covariance matrix of the injected object class in the *HS* plane. The segmentation is performed by thresholding the *pdf* (28) with a *T_h_* value. This threshold is obtained knowing that we want to segment the class O_i_ taking the background class B_i_ as reference, so, *T_h_* corresponds to the value of *pdf* (28) when: $d_{m}=\frac{1}{2}{\left(h_{\mathrm{iB}}-h_{\mathrm{iO}}\right)}^{T}{\left(\Sigma_{\mathrm{iO}}+\Sigma_{\mathrm{iB}}\right)}^{-1}\left(h_{\mathrm{iB}}-h_{\mathrm{iO}}\right)=\frac{1}{2}\text{tr}\left(M_{w}^{-1}M_{b}\right)$. Therefore, *T_h_* is given by:

(29) $$T_{h}=\frac{1}{2\pi {\left|\Sigma_{\mathrm{iO}}\right|}^{1/2}}\text{exp}\left\{-\frac{1}{4}\text{tr}\left(M_{w}^{-1}M_{b}\right)\right\}$$

The problems derived from the cyclical nature of the hue in the segmentations have been solved via software, using the convention introduced by Zhang and Wang [8].

In the evaluation, the same number of samples (seeds) for the object class (O) and the background (B) has been taken, *M* = *N*, in order to ensure that the difference between their statistical data is for intrinsic reasons, and not for differences in the sample space dimension. In the tests, the following data have been used: samples number: *M* = *N* = 50. Other tests with a higher number of seeds (*M* = *N* = 100, 200, 400, 800 and 1,600) have also been carried out, providing similar qualitative results in all of them, but with an increase in the iterative process computational cost. The increase of *θ* used in the algorithm shown in Figure 8: *Δθ* = 5°, interpolation interval *ΔΘ =* ±3*Δθ* and the weighting factor *k_h_* in (26) has been experimentally selected for each experiment, always fulfilling *k_h_* ∈ [0.75, 0.97]. In this stage, the experimental results have been quantified by means of the *FR* defined in (5). Table 1 shows the values of *FR* for 14 cases of the bank of images used in the tests.

**Figure 15.**
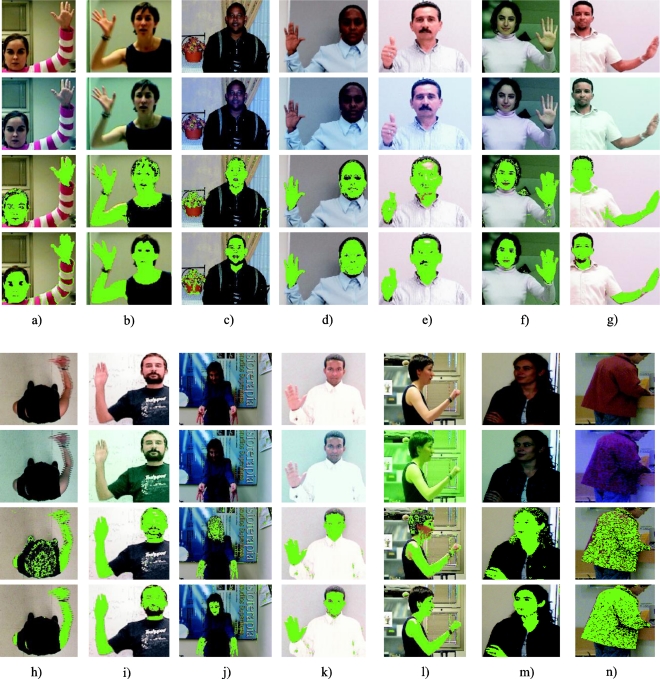
Segmentation results for objects in different environments.

Fourteen examples of segmentation can be seen in Figure 15 (figures a, b, c, d, e, f, g, h, i, j, k, l, m and n) that correspond with the 14 cases of *FR* calculated in Table 1. Four images are shown in each column (from up to down): the upper image is the original one (I), the second one is the coloured image (I_i_), the third one, the results of the segmentation of the original image (I segmentation), and the lower one, the results of the segmentation of the injected image (I_i_ segmentation). The segmented images show the object pixels in green colour. For the figures between Figure 15a and Figure 15m, the object class (O) is the skin, and for Figure 15n, the object class is a jacket.

As can be observed, our proposal to inject a colour vector allows the attainment of remarkable improvements in the segmentation process, even with a segmentation technique as studied and effective as the Gaussian classifier.

As a second phase of the experimental tests, and in order to quantify the improvement in the segmentation of the injected image with respect to the original image, an analysis, pixel by pixel, has been made, comparing with the manually segmented reference images for the 14 cases. The data generated in this analysis, without noise added, are shown in Table 2. The performance of the segmentation has been measured taking into account the classification Correct Detection Rate (*CDR*) and False Detection Rate (*FDR*) and the total Classification Rate (*CR*). *CDR* is the percentage of object pixels correctly classified, *FDR* is the percentage of background pixels incorrectly classified and *CR* is the total percentage of correctly classified pixels. Table 2 also shows the number (K) of Gaussians used by the GMM algorithm, the *FR* obtained by the statistics given by GMM for both classes, as well as the *k_h_* used for each image.

Table 3 shows the results of the comparison of the same images, but contaminated by additive zero-mean Gaussian noise. As can be seen, results obtained with the colour injection technique for both tests are better than those obtained using only a Gaussian classification.

As a third phase of the tests, an example of image sequence segmentation is presented. In this case, each frame illumination has been modified before the segmentation process, in order to verify the advantage of our proposal against illumination changes. The illumination is applied to each frame in a uniform way. A zero-mean Gaussian noise with standard deviation *n_p_* = 0.15*I_O_* was also added to the pixels of the images. Moreover, a sinusoidal time variation in the luminous intensity has been set up.

With this example, we try to show the improvements in the segmentation phase when the colour injection preprocessing step proposed in this paper is used before the segmentation. In this example, the GMM technique is used as an on-line segmentation technique (the same used in the off-line process). The original image I is identified by I*^k^* for each captured frame in time *kT* (*k* = 1, 2... and *T* = time between consecutive images), and its corresponding image after the colour injection, $I_{i}^{k}$, are segmented using the optimal class number obtained as a result of the off-line stage. In this case K_opt_ = 5.

For the images I*^k^* and $I_{i}^{k}$, the GMM segmentation process is applied recursively using the a priori probabilities, means and variances obtained in the images I*^k^*^−1^ and $I_{i}^{k-1}$, respectively. For the segmentation of the image $I_{i}^{k}$, the next steps are also added: (*a*) we obtain the pixels (seeds) in the *RGB* space of the object and background of the image $I_{i}^{k-1}$, (*b*) the vector **i_r_** is subtracted from them, (*c*) they are transformed to the *HSI* space, (*d*) the truncation process described in Section 4 is applied, and, finally, (*e*) the sets $O_{\mathrm{RGB}}^{k}$ and $B_{\mathrm{RGB}}^{k}$ are obtained. These steps (*a*, *b*, *c*, *d* and *e*) represent the block: “Obtaining seeds: Object (O) and Background (B)”, for recursive segmentation in the block-diagram of the Figure 1.

**Figure 16.**
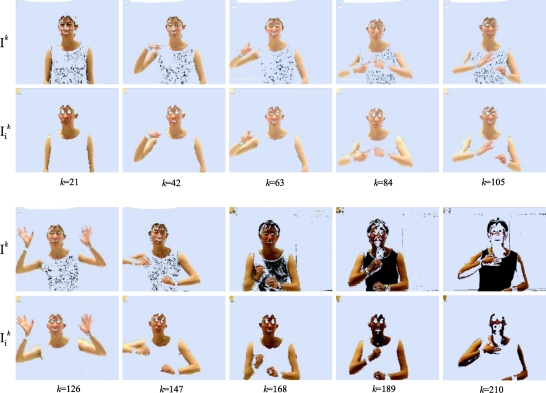
Segmentation results for 10 frames of an image sequence of a person generating sign language with big temporal illumination changes.

In image sequence segmentation, as this example, the iterative process (seen in Section 6) has been slightly modified in order to reduce the processing time and to increase the stability of the colour injection in time. The first modification, is to use $\theta_{\text{opt}}^{k-1}$ (*θ*_opt_ of the previous frame) as a starting point to obtain $\theta_{\text{opt}}^{k}$, thus reducing the search interval to: $\left[\theta_{\text{opt}}^{k-1}-\theta_{f},\;\theta_{\text{opt}}^{k-1}+\theta_{f}\right]$. In the example of Figure 16, we have fixed experimentally *θ_f_* = 12°, *Δθ* = 1° and *k_h_* = 0.91. The second modification in the iterative process is that the optimal colour vector to inject $i_{r}^{k}$ is obtained recursively, using for the calculation of $\theta_{\text{opt}}^{k}$ the following expression: $\theta_{\text{opt}}^{k}=k_{t}\theta_{\text{opt}}^{k}+\left(1-k_{t}\right)\theta_{\text{opt}}^{k-1}$, where *k_t_* ∈ [1, 0] is the constant fixed to obtain a proper smoothing of the evolution of the different parameters involved in the colour injection. *k_t_* has been fixed experimentally to 0.1.

The GMM technique is used in these tests, mainly to obtain a better adjustment of the K_opt_ Gaussians in each frame, and, therefore, to obtain the maximum quality in the object segmentation. Then, a reliable comparison of the segmentation quality between the segmentation of the images I and I_i_ in the time can be carried out, and the compensation effects in the segmentation against illumination changes, applying the colour injection or not, can be verified. However, as it is known, this technique may have a relatively high computational cost due to the convergence iterations of the EM algorithm, so, its use in video segmentation is sometimes limited. For the consecutive segmentation of an image sequence in real time, our proposal in this work is to track recursively the parameters that define each Gaussian: O and B, using the optimal estimation provided by the Kalman filter, tracking technique widely studied in the image processing field.

Figure 16 shows the results of the segmentation of the images I*^k^* and $I_{i}^{k}$ of the example sequence, for the frames captured in *k* = 21, 42, 63, 84, 105, 126, 147, 168, 189 and 210. The respective mean intensities of these frames are: $I_{m}^{21}=0.530$, $I_{m}^{42}=0.613$, $I_{m}^{63}=0.672$, $I_{m}^{84}=0.698$, $I_{m}^{105}=0.658$, $I_{m}^{126}=0.572$, $I_{m}^{147}=0.510$, $I_{m}^{168}=0.403$, $I_{m}^{189}=0.307$ and $I_{m}^{210}=0.224$.

However, if the variation of the parameters of the different classes of the scene in the image sequence is very small, that is, when the scene is relatively uniform in the time with small illumination changes, the colour injection can be carried out applying the same colour vector **i_r_** to each frame in the time *kT*, with no need to recalculate it. This is possible due to the illumination compensation effect previously mentioned in Section 7. In this sense, the segmentation can be carried out keeping the parameters of both Gaussians as fixed values in all the sequence. Then, the computational cost is noticeably reduced.

**Figure 17.**
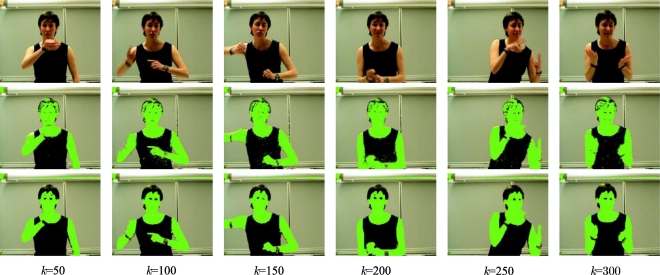
Segmentation results of 6 frames of an image sequence of a person generating sign language with small temporal illumination changes.

Figure 17 depicts the results of the segmentation of the images I*^k^* and $I_{i}^{k}$ corresponding to the instants: *k* = 50, 100, 150, 200, 250 and 300 of the previous example image sequence, but, this time, without recalculating the colour vector **i_r_** and with fixed parameters for both Gaussians. The objective of this example is to appreciate the improvement in the segmentation of the sequence of colour injected images, although the same colour vector **i_r_** is used in the injection.

In Figure 17, the upper row depicts the I*^k^* images, the central row shows the results of the segmentation without colour injection (I*^k^* segmentation), and the lower one contains the results of the segmentations after the colour injection proposed in this work ( $I_{i}^{k}$ segmentation). The segmented images show the object pixels in green colour. The segmentation process used in this phase has been used in the first stage of the experimental tests.

As a reference, the average execution time (*T_p_*) in Matlab of the on-line process for different *M* = *N* values is approximately: *T_p_* = 74.9 ms. for *N* = 50, *T_p_* = 80.0 ms. for *N* = 100, *T_p_* = 85.4 ms. for *N* = 200, *T_p_* = 95.9 ms. for *N* = 400, *T_p_* = 117.3 ms. for *N* = 800 and *T_p_* = 160.4 ms. for *N* = 1,600. The tests have been made with the following configuration: *θ_f_* *=* 12° in the recursive process, 10% of pixels segmented in the previous frame are used to obtain the $O_{\mathrm{RGB}}^{k}$ and $B_{\mathrm{RGB}}^{k}$ sets, and the image size is 346 × 421 pixels. The image size affects the execution time of the injection of **i_r_** to the original image, and the conversion of the injected image to the *HS* plane for its posterior segmentation. The tests have been carried out in a PC with a processor Intel Core 2 Duo with a 2.4 GHz frequency.

**Figure 18.**
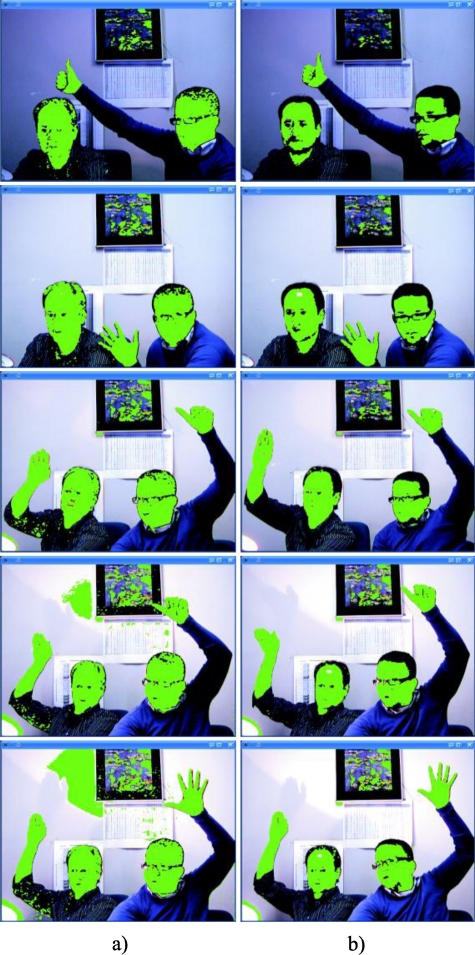
Results of the real-time segmentations with different illumination levels: (a) segmentation of original images captured directly from the camera, (b) segmentation of the images after the colour injection.

Finally, we show some results of the real-time segmentation of images captured in a scene with significative illumination changes. These results highlight again the advantages of using the colour injection proposal presented in this paper. Figure 18 depicts the comparative results in two columns: the left column (a) shows the segmented images without the colour injection, and in the right one (b) the images segmented after applying the colour injection.

The segmentation has been performed by thresholding the *pdf* of the skin class seen in (28), once all the classes have been obtained with the GMM algorithm. In this case, K = 10 predefined classes were used. In order to demonstrate the robustness to illumination changes, an incandescent light bulb has been used (that produces a hue change in the whole image that tends to yellow) to really change the illumination of the scenes. The different luminous Intensity levels have been quantified with the mean intensity of the image normalized between [0, 1]. The corresponding Intensity levels for the five images of each column of Figure 18, starting from the image above, are: I_1_ = 0.351, I_2_ = 0.390, I_3_ = 0.521, I_4_ = 0.565, I_5_ = 0.610.

In the performance of this last practical test, a PC with an Intel Quad Core Q6600 @ 2.4 GHz processor and 2 GB SDRAM @ 633 MHz memory has been used. Although it is a last generation PC with four processing cores (CPUs), our application has only used a single CPU. A Fire-Wire video camera with a 1/2” CCD sensor with a 640 × 480 spatial resolution and an image capture rate of 30 fps (*RGB* without compression) was used. The optic used is a C-Mount of 3/4” with a focal length of f = 12 mm. The different algorithms of our proposal (GMM, colour injection and segmentation) have been developed in C, under Linux OpenSuSE10.3 (×86_64) operating system. With this configuration, the average processing time of the on-line process (*T_p_*) is approximately 2 ms for N = 50.

## 9. Conclusions

A method to increase the separation between two classes in a pixel classification process has been proposed. The experimental results demonstrate that injecting colour in the captured image guarantees good results in maximizing the class separation, implying that class distributions adopt more Gaussian shapes, and, therefore, the segmentation of the desired object improves.

Its practical implementation results are simple and the process time is small. Even though the algorithm needs to calculate both class deviations in each iteration, these are easy to obtain, considering that classes are formed by a limited number of samples (*N*), the increase of *θ_i_* is not very small, *Δθ* = 1°, and the search interval is not very wide: *θ_f_* = 12°. This implies that calculations are relatively fast.

In this work, the expressions of interest to understand the vector’s behaviour in the *HSI* space have been demonstrated from the respective statistics in the *RGB* space. Moreover, the equations to convert directly from *YC_1_C_2_* colour space to *HSI* space have been obtained.

Finally, we should indicate that the images have been directly obtained from the classification process without any other auxiliary stage, such as morphological operations.

For future work, our research is currently focused on the injection of a vector **i_r_** with a non-zero mean, as a function of the intensity mean desired in the image, to increase the compensation of the effects caused by the illumination changes. We are also developing the hue and saturation dispersion model when the classes are translated all over the *HS* plane, using the *C_1_C_2_* plane, (similar to the hue and saturation deviation estimation that is made in [31] for the *HSI* space defined in [24]). This will diminish the processing time, because it will not be necessary to calculate the hue and saturation variances of both classes in each iteration. Finally, we are doing research into the class separation applying higher order transformations that imply scales and rotations of the classes in the *C_1_C_2_* plane. This could solve part of the intrinsic limitations of the colour injections for just adding the vector **i_r_**.

## Figures and Tables

**Table 1.** *FR* results for 14 cases in this work.

**Case**	***FR***	***FR* (Injected)**	**%Increase**

1	49.15	112.32	128.53
2	74.67	246.82	230.52
3	11.18	21.84	95.31
4	68.08	1,826.02	2,581.97
5	100.82	214.46	112.71
6	96.27	173.50	80.22
7	209.62	735.81	251.01
8	23.91	63.16	164.15
9	123.15	2,277.07	1,749.00
10	9.49	44.52	369.11
11	126.27	946.48	649.56
12	21.02	197.71	840.32
13	65.13	74.68	14.66
14	1.604	5.57	247.70

**Table 2.** Comparative analysis of the segmentations for the 14 example images shown in Figure 15, without noise added.

**Case**	Reference pixels^*^	***CDR* (%)**		***FDR* (%)**		***CR* (%)**		**Angle (°)**		**i_r_ vector**			***k_h_***	**K**	**Fisher Ratio**	
	
		**I**	**I_i_**	**I**	**I_i_**	**I**	**I_i_**	***θ***	***θ_i_***	**[*R***	***G***	***B*]**			***FR***	***FR_i_***

a	10,503	91	97	33	33	67	67	25	179	[−24	0	24]	0.97	5	23	39
b	18,340	94	97	22	10	78	90	32	116	[−23	4	18]	0.93	5	16	55
c	5,749	96	89	76	58	24	42	7	282	[−15	−4	19]	0.65	11	6	9
d	13,460	93	97	13	11	87	89	4	258	[−15	1	15]	0.65	5	6	84
e	10,097	84	97	26	10	74	90	23	246	[−26	−1	27]	0.87	5	47	157
f	12,666	97	97	47	24	53	76	56	179	[−4	−8	13]	0.9	7	15	122
g	13,775	98	91	28	13	72	87	18	171	[−31	14	16]	0.93	5	48	1,118
h	12,231	91	96	115	29	−15	71	14	158	[−21	6	15]	0.95	6	5	77
i	9,063	97	95	34	22	66	78	6	126	[−30	20	10]	0.93	5	11	139
j	12,497	89	97	100	55	0	45	20	167	[−13	2	10]	0.85	5	3	17
k	12,512	97	95	22	14	78	86	3	193	[−27	7	20]	0.95	5	19	176
l	23,102	69	94	59	17	41	83	17	23	[−39	44	−6]	0.87	10	5	24
m	20,176	99	96	31	12	69	88	26	56	[−16	15	1]	0.6	5	25	27
n	38,629	67	82	35	19	65	81	6	298	[−13	−19	33]	0.55	4	5	21

Average		90	94	46	23	54	77	18	175	[−21	6	15]	0.83	6	17	148

* Segmented Object Reference Image, I original image, Ii injected image

**Table 3.** Comparative analysis of the segmentations for the 14 example images shown in Figure 15, contaminated these images by additive zero-mean Gaussian noise.

**Case**	Reference Pixels^*^	***CDR* (%)**		***FDR* (%)**		***CR* (%)**		**Noise^†^(*σ*^2^)**	**Angle (°)**		**i_r_ vector**			***k****_h_*	**K**	**Fisher Ratio**	
		
		**I**	**I_i_**	**I**	**I_i_**	**I**	**I_i_**		***θ***	***θ****_i_*	**[*R***	***G***	***B*]**			***FR***	***FR****_i_*

a	10,503	51	76	86	52	14	48	0.8 × 10^−3^	2	74	[−20	2	18]	0.97	5	3	4
b	18,340	67	84	44	30	56	70	2.0 × 10^−3^	35	78	[−24	13	11]	0.93	5	4	10
c	5,749	89	74	140	117	−40	−17	0.5 × 10^−3^	2	27	[−15	−4	19]	0.65	7	4	5
d	13,460	62	70	42	38	58	62	1.0 × 10^−3^	12	272	[−20	0	21]	0.95	6	4	28
e	10,097	57	85	47	23	53	77	1.5 × 10^−3^	40	262	[−31	0	31]	0.95	5	4	17
f	12,666	48	60	70	51	30	49	0.5 × 10^−3^	158	202	[−5	−8	13]	0.9	7	3	6
g	13,775	79	84	30	26	70	74	1.5 × 10^−3^	10	256	[−30	0	30]	0.93	5	8	16
h	12,231	70	80	64	62	36	38	0.8 × 10^−3^	1	94	[−26	14	12]	0.95	8	8	17
i	9,063	77	92	42	25	58	75	1.5 × 10^−3^	128	265	[−27	−5	31]	0.93	5	5	13
j	12,497	64	75	115	74	−15	26	1.0 × 10^−3^	14	344	[−13	2	10]	0.85	10	3	5
k	12,512	89	89	33	21	67	79	1.0 × 10^−3^	1	291	[−20	−8	27]	0.9	6	5	13
l	23,102	80	81	90	29	10	71	0.5 × 10^−3^	12	14	[−49	75	−26]	0.87	7	5	7
m	20,176	62	76	52	38	48	62	1.3 × 10^−3^	19	20	[−9	26	−17]	0.88	5	2	6
n	38,629	49	90	72	40	28	60	1.0 × 10^−3^	11	43	[−23	34	−11]	0.6	4	4	17

Average		67	80	66	45	34	55	1.0 × 10^−3^	32	160	[−22	10	12]	0.88	6	4	12

* Segmented Object Reference Image, I original image, I_i_ injected image,

† Additive Gaussian noise: N(0, *σ*^2^)

## Appendix A

In this appendix, the relationships between the RGB, the HSI and YC1C2 spaces are shown.

Given a vector **r** = [*R G B*]*^T^* located in the *RGB* space, a vector **c′** = [*Y C_1_* *C_2_*]*^T^* in the *YC_1_C_2_* space can be calculated using the following expression [8,22,23]:

(A.1) $$\left[\begin{array}{l} Y\\ C_{1}\\ C_{2} \end{array}\right]=Q\left[\begin{array}{l} R\\ G\\ B \end{array}\right]$$

where **Q** is the space transformation matrix, given by:

(A.2) $$Q=\left[\begin{array}{ccc} 1/3 & 1/3 & 1/3\\ 1 & -1/2 & -1/2\\ 0 & \sqrt{3}/2 & -\sqrt{3}/2 \end{array}\right]$$

From (Equation A.1) the components *C_1_* and *C_2_* of the vector **c** = [*C_1_* *C_2_*]*^T^* are:

(A.3) $$C_{1}=R-1/2G-1/2B,\;C_{2}=\sqrt{3}/2G-\sqrt{3}/2B$$

From the last equation, the module (Chroma component, *C*) and angle, *H’*, of the vector **c** in the plane *C_1_C_2_* are given by:

(A.4) $$C=\left\|c\right\|={\left(C_{1}^{2}+C_{2}^{2}\right)}^{1/2}={\left(R^{2}+G^{2}+B^{2}-\mathrm{RG}-\mathrm{GB}-\mathrm{BR}\right)}^{1/2}$$

(A.5) $$H^{\prime }=\{\begin{array}{cc} \begin{array}{c} \alpha ,B\leq G\\ 2\pi -\alpha ,\;\text{otherwise} \end{array}; & \alpha ={\text{cos}}^{-1}\left(\frac{R-1/2G-1/2B}{{\left(R^{2}+G^{2}+B^{2}-\mathrm{RG}-\mathrm{GB}-\mathrm{BR}\right)}^{1/2}}\right) \end{array}$$

On the other hand, the components of a vector **h′** = [*H S I*]*^T^* in the *HSI* space [26] are given by:

(A.6) $$H=\{\begin{array}{ccc} \begin{array}{c} \gamma ,B\leq G\\ 2\pi -\gamma ,\;\text{otherwise} \end{array}; & & \gamma ={\;\text{cos}}^{-1}\left(\frac{R-1/2G-1/2B}{{\left(R^{2}+G^{2}+B^{2}-\mathrm{RG}-\mathrm{GB}-\mathrm{BR}\right)}^{1/2}}\right) \end{array}$$

(A.7) $$S=\left(1-\frac{3\text{min}\left(R,G,B\right)}{\left(R+G+B\right)}\right)$$

(A.8) I=(R+G+B)/3

We can observe that the angles of the vectors **c** = [C_1_ C_2_]^T^ and **h** = [H S]^T^ coincide (vectors superimposed on the *HS* plane). Therefore, it has been demonstrated that a vector **r** in the *RGB* space can be projected in the *HS* and *C_1_C_2_* planes with the same phase shift but a different module, that is: *H* = *H’* but *S* ≠ *C*. The relationship between *S* and *C* is shown the next appendix.

## Appendix B

In this appendix, the relationships between the statistics of vectors in *RGB* components, **r**, and its relationship with the components of their respective vectors, **h**, in the *HSI* space or *HS* plane are shown.

*Intensity (I)*: If *μ* is defined as the mean of the vector **r**, whose expression is:

(B.1) μ=(R+G+B)/3

Then, knowing the expression of the intensity component in the *HSI* space (Equation A.8), the intensity of the vectors **h**’ and **c**’ is given by:

(B.2) I=Y=μ

*Hue (H):* Using (Equation B.1), the expression of the variance of the vector **r** is given by:

(B.3) $$\sigma^{2}=\frac{2}{9}\left(R^{2}+G^{2}+B^{2}+\mathrm{RG}+\mathrm{GB}+\mathrm{BR}\right)$$

Relating the Chroma (*C*) expression (Equation A.4) with (Equation B.3), we can conclude:

(B.4) $$\left\|c\right\|=C={\left(C_{1}^{2}+C_{2}^{2}\right)}^{1/2}=\sqrt{9/2}\sigma$$

Then, the equations of the angles of **c** in the *C_1_C_2_* plane and **h** in the *HS* plane can be rewritten as:

(B.5) $$H=\{\begin{array}{cc} \begin{array}{c} \delta ,B\leq G\\ 2\pi -\delta ,\;\text{otherwise} \end{array}; & \delta ={\;\text{cos}}^{-1}\left(\frac{R-1/2G-1/2B}{\sqrt{9/2}\sigma }\right) \end{array}$$

Therefore, for any two vectors **r_1_**, **r_2_**, in *RGB* components and with similar deviations (*σ*), only six possible values of *H* exist in all the range of *H* in the *HS* plane where the two vectors overlap. This is fulfilled, independently of the intensity of each one, because they are uncorrelated. Since *C_1_* can be expressed only as a function of *σ* without *μ*(*I*), the previous equation (Equation B.5) shows the independence between the components *H* and *I*.

*Saturation (S):* The analysis of the saturation component should be made between the colour sectors of the *HS* plane, *i.e.*, (0−2π/3), (2π/3−4π/3) and (4π/3−2π), because these ranges delimit the three discontinuities of the saturation function. Knowing that *C_1_C_2_* plane has the same colour ranges that *HS* plane, considering the colour sector (0−2π/3) and supposing that **c** is within this sector, its angle can be expressed by any or the following expressions:

(B.6) $$H={\text{cos}}^{-1}\left(\frac{R-1/2G-1/2B}{\sqrt{9/2}\sigma }\right),\;\;H={\text{sin}}^{-1}\left(\frac{\sqrt{3}/2G-\sqrt{3}/2B}{\sqrt{9/2}\sigma }\right)$$

The *B* component is the minimum one for this colour sector, therefore, the saturation equation (Equation A.7) is given by:

(B.7) $$S=\frac{\left(R+G-2B\right)}{\left(R+G+B\right)}.$$

Multiplying by $2\sqrt{9/2}\sigma$ numerator and denominator of (Equation B.7), and applying (Equation B.1), the following expression is obtained:

(B.8) $$S=\frac{\sqrt{2}\sigma }{\mu }\left(\frac{1}{2}\frac{\left(R-1/2G-1/2B\right)}{\sqrt{9/2}\sigma }+\frac{\sqrt{3}}{2}\frac{\left(\sqrt{3}/2G-\sqrt{3}/2B\right)}{\sqrt{9/2}\sigma }\right)$$

Substituting (Equation B.6) in (Equation B.8), the equation of this colour sector saturation is obtained:

(B.9) $$S=\sqrt{2}\frac{\sigma }{\mu }\;\text{cos}\left(H-\pi /3\right).$$

Therefore, the general equation of the saturation is given by:

(B.10) $$S=\sqrt{2}\frac{\sigma }{\mu }f\left(H\right);\;\;f\left(H\right)=\{\begin{array}{l} \text{cos}\left(H-\pi /3\right);\;\;\left(0<H\leq 2\pi /3\right)\\ \text{cos}\left(H-\pi \right);\;\;\left(2\pi /3<H\leq 4\pi /3\right)\\ \text{cos}\left(H-5\pi /3\right);\;\;\left(4\pi /3<H\leq 2\pi \right) \end{array}.$$

where *f*(*H*) is a weighting function that takes values in the range from ½ to 1. This function *f*(*H*) generates a three lobe curve in the *HS* plane delimitated by the discontinuities corresponding to the three colour sectors of the plane: (0−2π/3), (2π/3−4π/3) and (4π/3−2π).

From (Equation B.10) we can conclude that the saturation component of a vector in the *HSI* space varies directly with the standard deviation of the *RGB* vector that produces it, and inversely proportionally with its mean.

In case we want to control the saturation of a colour keeping the same intensity, only the standard deviation (*σ*) of the *RGB* vector needs to be controlled, forcing its mean not to vary, *i.e.*,: *I* = constant. Therefore, relating (Equation B.4) with (Equation B.10) the following expression is obtained:

(B.11) S=KCf(H)=K‖c‖f(H)

where *K* = 3/(2*I*).

Equation (Equation B.11) represents the relationship between saturation *S* and the vector **c** associated in the *C_1_C_2_* plane. As we can observe, the saturation can be controlled varying the magnitude of the vector **c** (*C*), which is achieved by modifying the standard deviation (*σ*) of the vector **r**. Due to *H* is also function of *σ* (Equation B.5), the effect of controlling *S* by means of **c** is also determined by the weighting function *f*(*H*).

Performing some operations in (Equation B.10), a new form to express the saturation component from its components *C_1_* and *C_2_* is obtained. Therefore, (Equation A.5), (Equation B.2) and (Equation B.10) may be used to obtain new expressions for the space transformations from *YC_1_C_2_* to *HSI*, given by:

(B.12) $$H=\{\begin{array}{ccc} \begin{array}{c} \psi ,C_{2}\geq 0\\ 2\pi -\psi ,\;\text{otherwise} \end{array}; & & \psi ={\text{cos}}^{-1}\left(\frac{C_{1}}{{\left(C_{1}^{2}+C_{2}^{2}\right)}^{1/2}}\right) \end{array}$$

(B.13) $$S=\{\begin{array}{c} \frac{C_{1}+\sqrt{3}C_{2}}{3Y}\Rightarrow 0<H\leq 2\pi /3\\ -\frac{2C_{1}}{3Y}\Rightarrow 2\pi /3<H\leq 4\pi /3\\ \frac{C_{1}-\sqrt{3}C_{2}}{3Y}\Rightarrow 4\pi /3<H\leq 2\pi \end{array}$$

(B.14) I=Y.

## Appendix C

In this appendix, invariants of the mean vectors in the *C_1_C_2_* plane are shown.

The invariants of the mean vectors are determined by the mean vectors **c_1_** and **c_2_** in the *C_1_C_2_* plane, or directly by the mean vectors **r_1_** and **r_2_** in the *RGB* space, and have the property of keeping constant independently of the colour injection performed. In order to demonstrate it, we define a vector **d**, which represents the distance between them, whose expression is given by:

(C.1) $$d=c_{1}-c_{2}={\left[\begin{array}{cc} d_{C1} & d_{C2} \end{array}\right]}^{T}$$

where

(C.2) $$\begin{array}{ccc} d_{C1}=C_{11}-C_{12}=d_{R}-1/2d_{G}-1/2d_{G}, & & d_{C2}=C_{21}-C_{22}=\sqrt{3}/2d_{G}-\sqrt{3}/2d_{B} \end{array}$$

where *d_R_*, *d_G_* and *d_B_* represent the *R*, *G* and *B* components, respectively, of the difference of the vectors **r_1_** and **r_2_**, and (*C_1_*_1_, *C_2_*_1_) and (*C_1_*_2_, *C_2_*_2_) the components of the vectors **c_1_** and **c_2_**. If a vector **i_r_** = [Δ*_R_* Δ*_G_* Δ*_B_*]*^T^* is injected in the *RGB* space, the new difference vector (**d_i_**) between the injected vectors (**c_i1_** and **c_i2_**) is given by:

(C.3) $$d_{i}=c_{i1}-c_{i2}={\left[\begin{array}{cc} d_{\mathrm{iC}1} & d_{\mathrm{iC}2} \end{array}\right]}^{T}$$

where the *d_iC1_* and *d_iC2_* components are formed by the *d_iR_*, *d_iG_* and *d_iB_* components according to (Equation C.2), where the subscript “i” indicates they have already been injected with the respective component of **i_r_**. As *d_iR_* = (*R*_1_ + Δ*_R_*) − (*R*_2_ + Δ*_R_*) = *R*_1_ − *R*_2_ = *d_R_*, also *d_iG_* = *d_G_* and *d_iB_* = *d_B_*, therefore:

(C.4) $$d_{i}=d$$

In correspondence with **i_r_**, if a translation vector in the *C_1_C_2_* plane is defined, such as *i_c_* = [*Δ_C1_* *Δ_C2_*]*^T^*, the effect produced in the difference vector **d_i_**, when **i_c_** is added to the vectors **c_1_** and **c_2_**, is the same as the effect produced by **i_r_**, because *d_iC1_* = (*C_1_*_1_ + Δ*_C1_*) − (*C_1_*_2_ + Δ*_C1_*) = *d_C1_* and also *d_iC2_* = *d_C2_*, therefore, (Equation C.4) is fulfilled.

From (Equation C.4) we can conclude that this difference vector is not affected by the injection of the vector **i_r_** in the *RGB* space, that is, by the addition of **i_c_** in the *C_1_C_2_* plane, remaining as invariant factors both its magnitude and its orientation. Therefore, the invariants are: ‖**d**‖ and *φ*, whose expressions are given by:

(C.5) $$\begin{array}{ccc} \left\|d\right\|={\left(d_{C1}^{2}+d_{C2}^{2}\right)}^{1/2}, & & \varphi =\{\begin{array}{l} {\text{cos}}^{-1}\left(d_{C1}/\left\|d_{c}\right\|\right);\;d_{C2}\geq 0\\ 2\pi -{\text{cos}}^{-1}\left(d_{C1}/\left\|d_{c}\right\|\right);\;d_{C2}<0 \end{array} \end{array}$$

The angle *θ* formed between both vectors is obtained with:

(C.6) $$\text{cos}\theta =\frac{c_{1}^{T}.c_{2}}{\left\|c_{1}\right\|.\left\|c_{2}\right\|}$$

where:

(C.7) $$c_{1}^{T}.c_{2}=\left(R_{1}R_{2}+G_{1}G_{2}+B_{1}B_{2}\right)-\left(R_{2}G_{1}+R_{2}B_{1}+G_{2}R_{1}+G_{2}B_{1}+B_{2}R_{1}+B_{2}G_{1}\right)/2$$

Considering the covariance, *Cov*_12_, of the vectors **r_1_** and **r_2_** in the *RGB* space, and relating it with (Equation C.7), the following expression is obtained:

(C.8) $$Cov_{12}=\left(2/9\right)c_{1}^{T}.c_{2}$$

Knowing that the Chroma (*C*) expression of the vector **c** in the *C_1_C_2_* plane is:

(C.9) $$\left\|c\right\|=C=\sqrt{9/2}\sigma$$

Substituting (Equation C.9) and (Equation C.8) in (Equation C.6), we finally obtain the angle expression:

(C.10) $$\theta ={\text{cos}}^{-1}\left(CC_{12}\right)$$

where *CC_12_* is the correlation coefficient of the vectors **r_1_** and **r_2_**, whose expression is given by:

(C.11) $$CC_{12}=\left(Cov_{12}/\sigma_{1}\sigma_{2}\right)$$

We can conclude from (Equation C.11) that the angle between two vectors in the *HS* plane is the *arccos* of the correlation coefficient between both vectors in the *RGB* space, and from (Equation C.10) we conclude that this angle has a range between 0 and π radians.
